# Tendência Temporal da Mortalidade por Doenças Cardiovasculares: Estudo Ecológico das Regiões e Unidades da Federação do Brasil, 2010-2023

**DOI:** 10.36660/abc.20260087

**Published:** 2026-09-10

**Authors:** José Francisco Kerr Saraiva, Ana Clara Paixão Campos, Gisele O’Dwyer, Anna Carolina Pinho da Silva, Bruna Bornhofen Barros, Monique Alves Padilha, Fernanda Cristina da Silva de Lima, Catia Cristina Martins Oliveira

**Affiliations:** 1 Pontifícia Universidade Católica de Campinas Campinas SP Brasil Pontifícia Universidade Católica de Campinas, Campinas, SP – Brasil; 2 Centro de Integração de Dados e Conhecimentos para Saúde Fundação Oswaldo Cruz Salvador BA Brasil Centro de Integração de Dados e Conhecimentos para Saúde (CIDACS), Fundação Oswaldo Cruz, Salvador, BA – Brasil; 3 Escola Nacional de Saúde Pública Sérgio Arouca Fiocruz Rio de Janeiro RJ Brasil Escola Nacional de Saúde Pública Sérgio Arouca – ENSP/Fiocruz, Rio de Janeiro, RJ – Brasil; 4 Centro Universitário Serra dos Órgãos Teresópolis RJ Brasil Centro Universitário Serra dos Órgãos (UNIFESO), Teresópolis, RJ – Brasil; 5 Universidade Federal do Rio de Janeiro Instituto de Estudos em Saúde Coletiva Rio de Janeiro RJ Brasil Universidade Federal do Rio de Janeiro – Instituto de Estudos em Saúde Coletiva (IESC), Rio de Janeiro, RJ – Brasil; 6 Ministério da Saúde Secretaria de Informação e Saúde Digital Brasília DF Brasil Ministério da Saúde, Secretaria de Informação e Saúde Digital (SEIDIGI), Brasília, DF – Brasil; 7 Centro de Desenvolvimento de Tecnologias em Saúde Fundação Oswaldo Cruz Rio de Janeiro RJ Brasil Centro de Desenvolvimento de Tecnologias em Saúde (CDTS), Fundação Oswaldo Cruz – Rio de Janeiro, RJ – Brasil

**Keywords:** Doenças Cardiovasculares, Mortalidade, Estudos de Séries Temporais, Epidemiologia

## Abstract

**Fundamento:**

O óbito por doença cardiovascular (DCV) representa um marcador essencial para avaliar os progressos e limites das políticas públicas no Brasil, sobretudo em territórios mais vulneráveis.

**Objetivo:**

Analisar a mortalidade por DCV, doenças hipertensivas (DH), doenças isquêmicas do coração (DIC) e doenças cerebrovasculares (DCBV) segundo sexo, no Brasil, regiões e estados, entre 2010 e 2023.

**Métodos:**

Estudo de série temporal com dados de mortalidade obtidos do Departamento de Informática do Sistema Único de Saúde (DATASUS). As taxas foram padronizadas por idade e a tendência temporal avaliada pelo modelo de regressão *Joinpoint*, com cálculo do percentual de variação anual (APC, *Annual Percent Change*) e da Variação Percentual Anual Média (AAPC, *Average Annual Percent Change*) para todo o período. Considerou-se intervalo de confiança de 95%.

**Resultados:**

Entre 2010 e 2023, ocorreram 4.991.937 mortes por DCV no Brasil (52,50% em homens). Mortalidade por DCV reduziu em homens (Distrito Federal: -3,58%, p<0,001; Sul: -2,23%, p<0,001), mas aumentou no Amazonas (1,04%, p<0,01) e Amapá (1,54%, p<0,01). DIC cresceu em homens no Amapá (5,21%, p<0,001) e Roraima (2,61%, p=0,03). DH aumentou na Bahia (homens: 1,38%, p=0,03; mulheres: 1,50%, p=0,04) e Rio Grande do Sul (homens: 1,93%, p<0,001; mulheres: 1,86%, p<0,01). DCBV tem panorama de redução para a maioria dos estados. Mortalidade prematura por DH aumentou entre homens do Sul (+1,61%, p<0,001).

**Conclusão:**

No Brasil, apesar dos avanços na prevenção e tratamento das doenças cardiovasculares, as melhorias não foram equitativas: persistem fortes desigualdades regionais e socioeconômicas, com maiores taxas de mortalidade entre populações vulneráveis.

## Introdução

As doenças cardiovasculares (DCV) permanecem como um dos principais desafios para a saúde global, figurando como a principal causa de mortalidade e uma das maiores responsáveis por incapacidade e perda de anos de vida saudável no mundo desde 1990.^1,2^Este cenário é impulsionado por mudanças nos fatores de risco – destacando-se o índice de massa corporal, que apresenta maior associação com DCV^3^ – e pelo crescimento e envelhecimento populacional.^2^ Em 2022, as DCV causaram cerca de 19,8 milhões de óbitos em todo o mundo, um terço do total global, resultando em uma perda de 396 milhões de anos de vida.^2^ Um dado relevante é que cerca de 34% dessas mortes foram prematuras (em pessoas de 30 a 69 anos), sendo consideradas amplamente evitáveis mediante intervenções já disponíveis, como o controle de fatores de risco.^1-5^

No Brasil, desde 1990, as doenças cardiovasculares lideram as estatísticas de mortalidade entre as doenças crônicas não transmissíveis (DCNT).^6^ Dentre elas, a doença arterial coronariana permanece como a principal causa de óbitos, seguida pelo acidente vascular cerebral (AVC).^6^ Essas doenças e suas complicações têm elevado impacto na perda de produtividade no trabalho e na redução da renda familiar, atingindo com maior intensidade as populações socioeconomicamente vulneráveis, cujo acesso limitado a serviços de saúde de qualidade aprofunda o ciclo de disparidades.^7^

Além dos fatores individuais, o território constitui dimensão central na produção das desigualdades em saúde no Brasil. O país apresenta heterogeneidades regionais históricas em renda, escolaridade, infraestrutura urbana e oferta de serviços de saúde, que se refletem nos indicadores sociais e sanitários.^8^ Estudos apontam que residentes de regiões menos desenvolvidas apresentam maior exposição a vulnerabilidades socioeconômicas e piores indicadores de saúde.^9,10^

No âmbito das políticas públicas, em 2011 o Ministério da Saúde lançou o Plano de Ações Estratégicas para o Enfrentamento das Doenças Crônicas Não Transmissíveis no Brasil, 2011-2022, com o objetivo de promover o desenvolvimento e a implementação de políticas públicas efetivas, integradas, sustentáveis e baseadas em evidências para a prevenção e o controle das DCNT. Além disso, foi implementada em 2022 a Estratégia de Saúde Cardiovascular na Atenção Primária à Saúde, alinhada à iniciativa global HEARTS (OMS/OPAS), visando fortalecer a detecção precoce e o controle de fatores de risco como hipertensão e diabetes. Contudo o avanço dessas políticas enfrenta desafios sociais, organizacionais e econômicos dificultando sua sustentabilidade e impacto nos indicadores de saúde.

Diante desse panorama, a análise desagregada da mortalidade por regiões e Unidades da Federação (UF) torna-se um marcador estratégico para avaliar a efetividade das políticas públicas que vêm sendo desenvolvidas no país, sobretudo devido às profundas iniquidades socioeconômicas e estruturais, contribuindo para identificar disparidades na expectativa de vida e nas causas de óbito, direcionar recursos de forma mais justa e prevenir mortes evitáveis.

Considerando a alta carga de mortalidade imposta pelas DCV e seus significativos custos diretos (hospitalização e procedimentos) e indiretos (perda de produtividade e falecimento prematuro), o presente estudo teve como objetivo analisar a tendência temporal das taxas de mortalidade por DCV padronizadas por idade e seus principais subgrupos – doenças hipertensivas (DH), doenças isquêmicas do coração (DIC) e doenças cerebrovasculares (DCBV) – no Brasil, regiões e Unidades da Federação, no período de 2010 a 2023.

## Métodos

### Desenho de estudo

Estudo ecológico de série temporal, com análise da mortalidade por DCV no Brasil, suas regiões e Unidades da Federação, conforme sexo, no período de 2010 a 2023, utilizando-se os óbitos registrados por local de residência como unidade de análise.

### Fonte de informação

Os dados sobre as causas da morte foram obtidos no site do Sistema de Informações sobre Mortalidade (SIM), mantido pelo Departamento de Informática do Sistema Único de Saúde (DATASUS) do Ministério da Saúde. Foram incluídos os registros de óbitos por DCV, definidos como a causa básica sob os códigos I00-I99 (Doenças do Aparelho Circulatório) da Décima Revisão da Classificação Estatística Internacional de Doenças e Problemas Relacionados à Saúde (CID-10). A análise focou também em três subgrupos de relevância para a saúde pública: DH (I10–I15), DIC (I20–I25) e DCBV (I60–I69).

### Análise estatística

As estimativas anuais de população residente no Brasil, suas regiões e UF foram obtidas no site do DATASUS, que utiliza dados oficiais do Censo do Instituto Brasileiro de Geografia e Estatística (IBGE) para o ano de 2010, juntamente com projeções intercensitárias, calculadas por meio de ajuste de uma função polinomial do 3º grau.^11^

Foram utilizadas as variáveis sexo (masculino e feminino), faixa etária em anos (< 20 anos (excluindo o 20), 20 a 29 anos, 30 a 39 anos, 40 a 49 anos, 50 a 59 anos, 60 a 69 anos e ≥70 anos), raça/cor (branca, preta, parda, amarela e indígena), escolaridade em anos (nenhuma, 1 a 3 anos, 4 a 7 anos, 8 a 11 anos), região de residência (Norte; Nordeste; Sul; Sudeste; Centro-Oeste), os 26 estados brasileiros (Acre, Alagoas, Amazonas, Amapá, Bahia, Ceará, Espírito Santo, Goiás, Maranhão, Minas Gerais, Mato Grosso do Sul, Mato Grosso, Pará, Paraíba, Pernambuco, Piauí, Paraná, Rio de Janeiro, Rio Grande do Norte, Rondônia, Rio Grande do Sul, Santa Catarina, Sergipe, São Paulo, Tocantins) e o Distrito Federal. Foi calculada a taxa de mortalidade, por 100 mil hab., padronizada por idade considerando as variáveis sexo, faixa etária, ano do óbito (entre 2010 e 2023), região e Unidade da Federação de residência.

O estudo conduziu inicialmente análises descritivas das variáveis sociodemográficas considerando o número e a proporção de óbitos por DCV no período de 2010 a 2023. As taxas de mortalidade foram padronizadas por idade pelo método direto, tomando-se como referência a população padrão mundial proposta por Segi.^12^ Este procedimento é essencial para minimizar o viés causado pelas variações na distribuição etária, isolando o efeito real do risco de morte por DCV. Para o cálculo da taxa de mortalidade padronizada, os dados preenchidos como ignorados foram excluídos das análises.

As tendências temporais foram estimadas pelo modelo de regressão *Joinpoint*. Esta metodologia tem como objetivo identificar o modelo que melhor descreve o comportamento das taxas ao longo do tempo, por meio de segmentos de reta conectados, com ou sem “pontos de inflexão” (*joinpoints*). Nessa abordagem, as taxas de mortalidade (variável dependente) foram transformadas em escala logarítmica, enquanto os anos do período estudado foram considerados como variável independente.^13^

A escolha do modelo final foi baseada em testes de permutação de Monte Carlo, adotando-se o que apresentasse o maior número de *joinpoints* com significância estatística (p<0,05).^13^ Após a definição do modelo selecionado, calculou-se a Variação Percentual Anual (APC - *Annual Percent Change*) e o Percentual de Variação anual Médio (AAPC – *Average Annual Percent Change*), com seus respectivos intervalos de confiança e valor de p. Os valores de AAPC aqui apresentados descrevem a direção (aumento, redução, estabilidade) e a intensidade das mudanças observadas ao longo do tempo.^13,14^ Foram adotados os seguintes critérios metodológicos: série histórica mínima de 10 anos e exclusão de valores zero na série temporal, visto que o cálculo das APCs e AAPCs utiliza transformação logarítmica, tornando o logaritmo de zero matematicamente indefinido, o que poderia comprometer a análise com erros. As análises estatísticas foram conduzidas nos softwares R (versão 4.5.1)^15^ e Joinpoint Regression Program (versão 5.4.0),^16^ sendo que a construção das tabelas e as figuras foram desenvolvidas no Microsoft Excel.

O estudo não necessitou de aprovação ética, uma vez que utilizou dados públicos de mortalidade sem identificação dos participantes.

## Resultados

De 2010 a 2023, ocorreram 4.991.937 óbitos por DCV no Brasil, sendo a maior parte no sexo masculino (52,50%) (Tabela 1). Dentre esses óbitos, as três principais doenças cardiovasculares foram responsáveis por uma parcela significativa: Doença Isquêmica do Coração causou 1.561.034 óbitos (aproximadamente 31,30% de todos os óbitos por DCV), Doença Cerebrovascular levou a 1.418.725 óbitos (aproximadamente 28,40%) e Doença Hipertensiva resultou em 749.392 óbitos (aproximadamente 15%). Juntos, esses três grupos representaram quase três quartos de todos os óbitos relacionados a DCV durante esse período (Figura central). A proporção de óbitos aumentou progressivamente com a idade em ambos os sexos. Os homens apresentaram percentuais mais elevados na maioria das faixas etárias, exceto entre indivíduos com 70 anos ou mais, onde as mulheres predominaram (68,60%). Mulheres brancas representaram 54,35% dos óbitos. As maiores proporções de óbitos em mulheres ocorreram no Sudeste (47,26%) e Nordeste (26,40%) e a baixa escolaridade (“Nenhuma” e “1 a 3 anos”) também concentrou maiores percentuais de óbitos entre mulheres (47,77%) (Tabela 1).

**Tabela 1:** – Caracterização sociodemográfica dos óbitos por doenças cardiovasculares no Brasil, 2010-2023 (n=4.991.937)

Variável	Masculino n=2.620.654 (52,50%)	Feminino n=2.370.766 (47,49%)	Ignorado n=517 (0,01%)
	n (%)	n (%)	n (%)
**Faixa etária**			
< 20 anos	11.773 (0,45)	8.969 (0,38)	4 (0,77)
20 a 29 anos	21.430 (0,82)	12.507 (0,53)	2 (0,39)
30 a 39 anos	57.266 (2,19)	35.997 (1,52)	10 (1,93)
40 a 49 anos	155.738 (5,94)	101845 (4,30)	11 (2,13)
50 a 59 anos	355.198 (13,55)	209440 (8,83)	39 (7,54)
60 a 69 anos	575.369 (21,96)	373.668 (15,76)	70 (13,54)
70+ anos	1.438.467 (54,89)	1.626.386 (68,60)	246 (47,58)
Ignorado	5.413 (0,21)	1.954 (0,08)	135 (26,11)
**Raça/cor**			
Branca	1.322.820 (50,48)	1.288.530 (54,35)	72 (13,93)
Preta	236.083 (9,01)	201.989 (8,52)	23 (4,45)
Parda	954.325 (36,42)	780.702 (32,93)	113 (21,86)
Amarela	15.563 (0,59)	14.353 (0,61)	4 (0,77)
Indígena	5.119 (0,20)	4.506 (0,19)	8 (1,55)
Ignorado	86.744 (3,31)	80.686 (3,40)	297 (57,45)
**Escolaridade (anos)**			
Nenhuma	425.034 (16,22)	549.058 (23,16)	45 (8,70)
1 a 3 anos	632.581 (24,14)	583.407 (24,61)	49 (9,48)
4 a 7 anos	564.843 (21,55)	450.234 (18,99)	27 (5,22)
8 a 11 anos	374.178 (14,28)	260.483 (10,99)	21 (4,06)
12 anos e mais	137.063 (5,23)	85.157 (3,59)	10 (1,93)
Ignorado	486.955 (18,58)	442.427 (18,66)	365 (70,60)
**Região**			
Norte	147.846 (5,64)	110.403 (4,66)	38 (7,35)
Nordeste	678.805 (25,90)	625.858 (26,40)	152 (29,40)
Centro-Oeste	178.827 (6,82)	141.063 (5,95)	49 (9,48)
Sudeste	1.216.092 (46,40)	1.120.534 (47,26)	240 (46,42)
Sul	399.084 (15,23)	372.908 (15,73)	38 (7,35)

**Figura Central:**
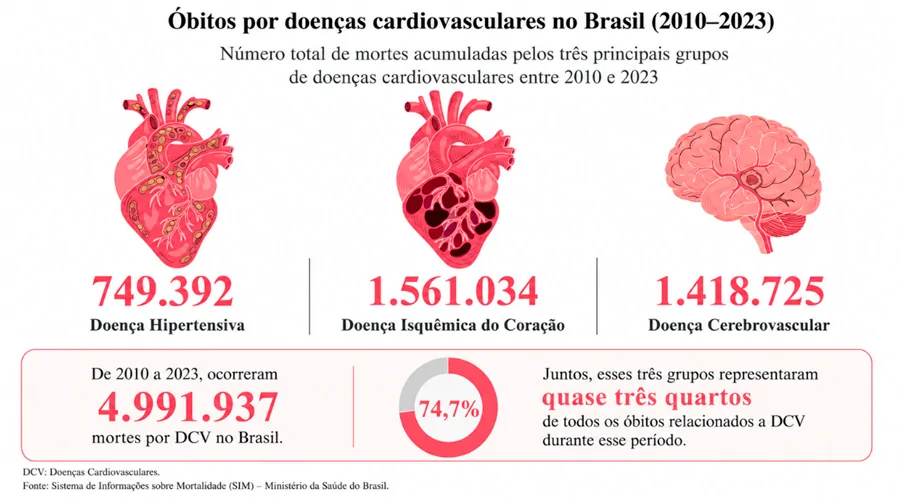
: Tendência Temporal da Mortalidade por Doenças Cardiovasculares: Estudo Ecológico das Regiões e Unidades da Federação do Brasil, 2010-2023

A análise dos segmentos temporais para DCV e os subgrupos (Tabelas S1 a S4) revelou maior oscilação (mais *joins*) para DCV na região Norte; para DCBV no Norte e Sudeste; para DH no Sudeste (3 segmentos) e Centro-Oeste; e para DIC no Norte, Nordeste e Centro-Oeste (3 segmentos para mulheres). Isso revela padrões de instabilidade que variam conforme a causa e a região. Na análise segmentada por região para a população de 30 a 69 anos (Tabela S5), observaram-se padrões distintos entre os agravos, com impacto evidente do período pandêmico nas trajetórias. DCV e DCBV apresentaram quedas até 2019-2020 em todas as regiões, lideradas pelo Sul e Sudeste, seguidas de estabilização ou leve reversão no período pandêmico. DH exibiu trajetória mais irregular, com destaque para o Sudeste (queda até 2015, aumento até 2021 e nova queda) e para os homens do Sul, que tiveram aumento no período de 2019-2023 (+5,27%). DIC foi o agravo mais estável e favorável, com quedas generalizadas, exceto pelo aumento entre homens do Norte do país de 2017 a 2023 (+1,18%).

As análises de tendência a seguir basearam-se nos valores de AAPC do modelo de regressão *Joinpoint*.

### Mortalidade por doença cardiovascular

Na Figura 1, observou-se queda significativa da mortalidade por DCV no Brasil (homens: -1,52%; p<0,001 e mulheres: -2,01%; p<0,001), com destaque para a região Sul, que apresentou as maiores reduções (-2,23%; p<0,001 e -2,63%; p<0,001, respectivamente). Em contraste, a região Norte revelou situações opostas: enquanto o Amapá registrou aumento para ambos os sexos (+1,54%; p<0,01 para homens e +2,05%; p=0,03 para mulheres) e o Amazonas apenas para os homens (+1,04%; p<0,01), estados como Rondônia e Tocantins se destacaram por expressivos declínios na população feminina (-1,68%; p<0,001 e -2,29%; p<0,001, respectivamente) — diferenças que reforçam a heterogeneidade regional no território brasileiro.

**Figura 1:**
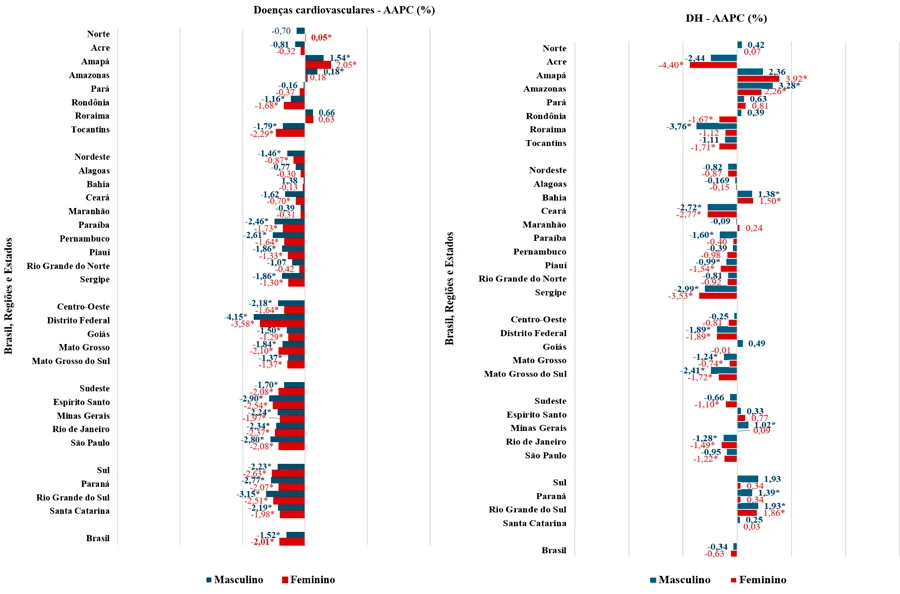
– Tendência das taxas de mortalidade por Doenças Cardiovasculares e Doenças Hipertensivas padronizada por idade (por 100 mil habitantes), segundo sexo, Brasil, regiões, estados e Distrito Federal, 2010 – 2023. *AAPC estatisticamente significativo (p<0,05).

### Mortalidade por doenças hipertensivas

Ainda na Figura 1, a análise da mortalidade por DH revelou padrões igualmente heterogêneos. Em nível regional, apenas o Sudeste apresentou queda significativa para as mulheres (-1,10%; p<0,01), enquanto nas demais regiões e no Brasil a tendência foi de estabilidade para ambos os sexos. E contraste com o padrão regional predominantemente estável, alguns estados exibiram variações expressivas. Na região Norte, destacaram-se aumentos no Amazonas (homens: +3,28%; p<0,01 e mulheres: +2,26%; p<0,01) e Amapá (mulheres: +3,92%; p=0,04), contrastando com quedas no Acre (mulheres: -4,40%; p=0,04), Roraima (homens: -3,76%; p=0,01), Rondônia (mulheres: -1,67%; p=0,01) e Tocantins (mulheres: -1,71%; p=0,03). No Nordeste, diferentemente da maioria dos estados, a Bahia exibiu aumento para ambos os sexos (+1,38%; p=0,03 em homens e +1,50%; p=0,04 em mulheres). No Centro-Oeste, predominaram quedas, especialmente entre homens do Distrito Federal (-1,89%; p=0,01) e Mato Grosso do Sul (-2,41%; p=0,01). No Sudeste, o Rio de Janeiro se destacou com redução para ambos os sexos (-1,28%; p<0,001 em homens e -1,49%; p<0,001 em mulheres). Por fim, na região Sul, observou-se aumento mais expressivo no Rio Grande do Sul (homens: +1,93%; p<0,001 e mulheres: +1,86%; p<0,01).

### Mortalidade por doenças isquêmicas do coração

Na Figura 2, a tendência da mortalidade por DIC revelou um contraste nacional entre os sexos: aumento entre homens e redução entre mulheres. Na região Norte, o padrão geral foi de elevação, puxado pelo crescimento masculino. Os casos em destaque foram os aumentos no Amapá (+5,21; p<0,001) e Roraima (+2,61; p=0,03) entre homens, e no Pará entre mulheres (+1,14; p<0,001). No Nordeste, a redução restrita às mulheres escondeu heterogeneidade interna: Alagoas registrou um dos maiores aumentos (+2,62; p=0,01) na população feminina do Brasil, contrastando com as expressivas quedas observadas em Pernambuco (-3,64; p<0,001). No Centro-Oeste, os declínios foram moderados e restritos a Mato Grosso e Mato Grosso do Sul. Já o Sudeste apresentou reduções significativas e generalizadas para ambos os sexos, com Rio de Janeiro (homens: -2,84%; p<0,001 e mulheres: -3,71%; p<0,001) liderando as quedas — estas entre as mais acentuadas do país. O Sul, por sua vez, configurou-se como a região de maior redução nacional, com destaque para as mulheres do Paraná (-4,58%; p<0,001), que atingiram os maiores declínios entre todas as regiões.

**Figura 2:**
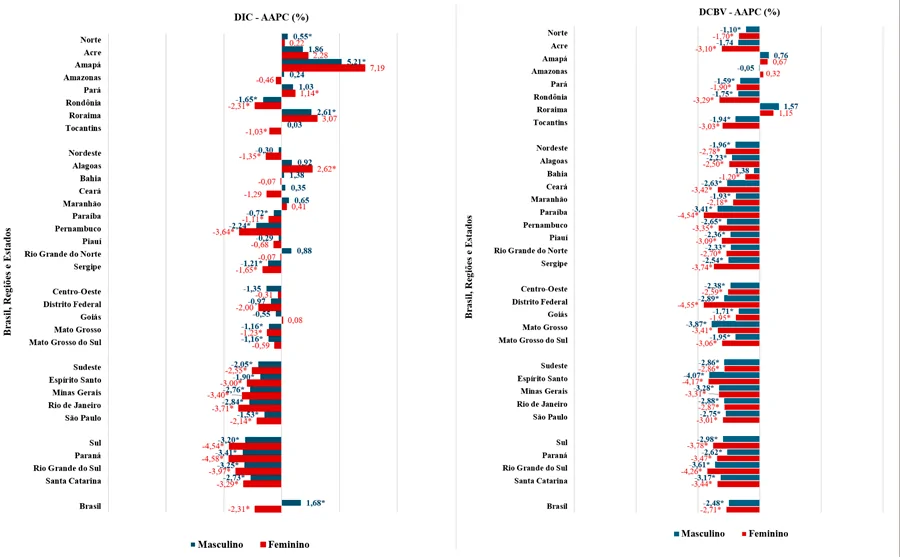
– Tendência das taxas de mortalidade por doenças isquêmicas do coração (DIC) e doenças cerebrovasculares (DCBV) padronizada por idade (por 100 mil habitantes), segundo sexo, Brasil, regiões, estados e Distrito Federal, 2010 – 2023. *AAPC estatisticamente significativo (p<0,05).

### Mortalidade por doenças cerebrovasculares

Para as DCBV (Figura 2), observou-se queda generalizada e significativa nas tendências de mortalidade para o Brasil e todas as regiões, em ambos os sexos — com as mulheres apresentando reduções mais expressivas. A região Sul liderou as quedas, enquanto o Norte, apesar da tendência favorável, exibiu os declínios mais modestos do país. Na Região Norte, observa-se um cenário em que a maioria dos estados apresentou queda nas tendências das taxas de mortalidade, especialmente Rondônia (-1,75%; p<0,01 para homens e -3,29%; p<0,001 para mulheres) e Tocantins (-1,94%%; p<0,001 para homens e -3,03%%; p<0,001 para mulheres). No Nordeste, exceto na população masculina da Bahia, todos os outros estados apresentaram tendência de queda na taxa de mortalidade por DCBV em ambos os sexos. Todos os estados do Centro-Oeste mostraram declínio das taxas de mortalidade. O Distrito Federal teve a maior redução entre as mulheres (-4,55%; p<0,001) e o Mato Grosso a maior queda entre os homens (-3,87%; p<0,001). No Sudeste, todos os estados reduziram as taxas de mortalidade para ambos os sexos, com o Espírito Santo (-4,07%; p<0,001 para homens e -4,17%; p<0,001 para mulheres) liderando as quedas mais acentuadas do país. Todas as unidades federativas do Sul do país apresentaram redução significativa na taxa de mortalidade, com destaque para a população feminina do Rio Grande do Sul (-4,26%; p<0,001).

### Mortalidade na população de 30 a 69 anos

A mortalidade prematura por DCV (Tabela 2) reduziu no Brasil, com quedas mais acentuadas entre mulheres (-2,19%), lideradas pelas regiões Sul (-2,77%) e Centro-Oeste (-2,62%). Para DH, a queda nacional foi significativa apenas entre mulheres (-1,56%), com destaque para O Centro-Oeste, Sudeste e Norte. Na DIC, a redução foi significativa em ambos os sexos, porém quase duas vezes maior entre mulheres (-2,58% vs. -1,77% nos homens), com o Sul liderando os declínios. Na DCBV, as quedas também foram significativas para ambos os sexos, mais pronunciadas entre mulheres (-2,87%), com maior declínio feminino no Sul e masculino no Sudeste. Em todas os grupos de causas, as mulheres apresentaram reduções superiores às dos homens em praticamente todas as regiões.

**Tabela 2:** – Tendência da taxa de mortalidade prematura (30 a 69 anos) por doenças cardiovasculares e subgrupos – DH, DIC e DCBV - padronizada por idade (por 100 mil habitantes), segundo sexo, Brasil, regiões, 2010 – 2023

Região		Masculino				Feminino			
		DCV	DH	DIC	DCBV	DCV	DH	DIC	DCBV
Norte	AAPC (IC95%)	-0,47* (-0,81 a -0,22)	-0,04 (-1,14 a 1,07)	0,14 (-0,54 a 0,84)	-1,24* (-1,67 a -0,75)	-1,51* (-1,97 a -1,06)	-1,34* (-2,58 a -0,09)	-1,18* (-1,76 a -0,59)	-2,41* (-3,02 a -1,79)
Nordeste	AAPC (IC95%)	-0,61* (-0,99 a -0,29)	-0,67 (-1,89 a 0,56)	-0,39* (-0,73 a -0,01)	-1,53* (-2,15 a -1,17)	-2,03 * (-2,44 a -1,63)	-1,65 (-3,46 a 0,69)	-1,76* (-2,66 a -1,16)	-2,97* (-3,41 a -2,65)
Centro-Oeste	AAPC (IC95%)	-1,87* (-2,23 a -1,52)	-1,24 (-2,69 a 0,20)	-1,16* (-1,63 a -0,69)	-2,55* (-3,09 a -2,11)	-2,62* (-3,29 a -2,11)	-2,53* (-3,54 a -1,50)	-1,9 * (-2,53 a -1,27)	-2,91* (-3,57 a -2,43)
Sudeste	AAPC (IC95%)	-1,63* (-2,09 a -1,24)	-1,24* (-1,99 a -0,54)	-2,02* (-2,45 a -1,59)	-2,98* (-3,29 a -2,54)	-2,39 * (-2,82 a -1,96)	-2,38* (-3,26 a -1,60)	-2,59* (-3,15 a -2,02)	-2,85* (-3,38 a -2,50)
Sul	AAPC (IC95%)	-2,05* (-2,59 a -1,66)	1,61* (0,71 a 2,42)	-3,25* (-3,82 a -2,69)	-2,69* (-3,53 a -2,07)	-2,77* (-3,51 a -2,19)	-0,53 (-1,85 a 0,97)	-4,04* (-4,73 a -3,35)	-3,04* (-3,87 a -2,46)
Brasil	AAPC (IC95%)	-1,47* (-1,81 a -1,18)	-0,84 (-1,88 a 0,10)	-1,77* (-2,15 a -1,38)	-2,3* (-2,75 a -1,98)	-2,19* (-2,61 a -1,89)	-1,56* (-2,49 a -0,41)	-2,58* (-3,08 a -2,07)	-2,87* (-3,23 a -2,62)

IC 95%: intervalo de confiança de 95%; AAPC: percentual de variação anual médio; DCV: doenças cardiovasculares; DH: doenças hipertensivas; DIC: doenças isquêmicas do coração; DCBC: doenças cerebrovasculares. *estatisticamente significativo.

## Discussão

Neste estudo, observou-se maior proporção de óbitos entre indivíduos com menor escolaridade, evidenciando que condições socioeconômicas desfavoráveis permanecem associadas a piores desfechos cardiovasculares.^17^ Evidências de outras pesquisas demonstram que baixa escolaridade está relacionada à maior exposição a fatores de risco cardiovasculares, incluindo alimentação inadequada, menor prática de atividade física, piores condições de moradia, maior prevalência de obesidade e hipertensão arterial, além de barreiras de acesso e continuidade do cuidado nos serviços de saúde.^18,19^No Brasil, a mortalidade cardiovascular deve ser compreendida não apenas como resultado de fatores biomédicos individuais, mas também como expressão das desigualdades sociais e estruturais historicamente presentes no país^19,20^— onde a sua persistência reforça o papel dos determinantes sociais da saúde nessa carga. Além da escolaridade, a distribuição dos óbitos segundo raça/cor reforça a relevância das iniquidades raciais na mortalidade cardiovascular, ainda que parte dos registros apresente informação ignorada, especialmente em populações historicamente vulnerabilizadas. Estudos nacionais demonstram que a população negra apresenta maior exposição a fatores de risco cardiovasculares, bem como expressivas dificuldades de acesso ao cuidado longitudinal em saúde.^20-28^ Importante ressaltar que a elevada proporção de registros ignorados em raça/cor, observada neste estudo, evidencia limitações persistentes na qualidade da informação, sinalizando a necessidade de maior rigor no seu preenchimento.

Sob a perspectiva da análise de tendência, os achados deste estudo demonstram que, embora o Brasil tenha apresentado redução global da mortalidade por DCV entre 2010 e 2023, essa transição epidemiológica ocorreu de forma heterogênea entre regiões, principalmente quando analisadas segundo sexo e subgrupos de doenças cardiovasculares. De forma geral, as reduções mais expressivas observadas nas regiões Sul, Sudeste e Centro-Oeste contrastam com tendências menos favoráveis registradas em estados das regiões Norte e Nordeste, sugerindo que os avanços nacionais coexistem com persistentes desigualdades estruturais, sociais e territoriais no acesso à prevenção, diagnóstico e tratamento das DCV. Esses resultados são consistentes com outros estudos nacionais e internacionais que demonstram redução progressiva da mortalidade cardiovascular em vários países, nas últimas décadas, porém acompanhada de importantes disparidades intra e inter-regionais.^23-28^

As diferenças entre homens e mulheres também merecem destaque. A mortalidade cardiovascular permaneceu, sistematicamente, mais elevada entre os homens, padrão já descrito na literatura nacional e internacional.^18,29^ Esse comportamento pode refletir menor procura por cuidado preventivo, menor adesão terapêutica e maior prevalência de fatores comportamentais de risco nesse grupo, como tabagismo, consumo abusivo de álcool e inatividade física no lazer.^18,19^ Entre as mulheres, embora a mortalidade geral tenha sido menor, as DCV frequentemente se apresentam com manifestações clínicas atípicas e menor reconhecimento diagnóstico, o que pode retardar o manejo adequado e contribuir para piores desfechos em idades mais avançadas.^29,30^ Esses achados reforçam a necessidade de estratégias preventivas e assistenciais, principalmente no âmbito da atenção primária à saúde, e que sejam sensíveis às diferenças de gênero ao longo do curso de vida.

Ao analisar os subgrupos específicos de DCV, observou-se redução importante da mortalidade por DIC e DCBV, especialmente nas regiões Sul e Sudeste. Esses resultados são consistentes com avanços observados na organização da linha de cuidado cardiovascular e cerebrovascular no Brasil, incluindo maior disponibilidade de terapias farmacológicas efetivas, ampliação de protocolos clínicos, expansão de unidades especializadas e incorporação gradual de tecnologias assistenciais, como telemedicina e sistemas de regulação do cuidado.^30-32^

Em contraste, a mortalidade por DH apresentou tendência de estabilidade ou aumento em alguns estados, particularmente nas regiões Norte e Nordeste sugerindo que, embora o país tenha avançado no manejo dos eventos cardiovasculares agudos, persistem desafios relacionados ao controle longitudinal da hipertensão arterial e ao manejo contínuo dos fatores de risco cardiovasculares.^33^

A hipertensão arterial permanece como um dos principais fatores de risco modificáveis para DCV e AVC, exigindo estratégias sustentadas de rastreamento, monitoramento clínico e adesão terapêutica ao longo do tempo.^33^ Nesse contexto, a atenção primária à saúde desempenha papel central na coordenação do cuidado, no acompanhamento longitudinal e na prevenção das complicações cardiovasculares, sobretudo em populações socialmente vulneráveis.

Dados da Pesquisa Nacional sobre Acesso, Utilização e Promoção do Uso Racional de Medicamentos (PNAUM) demonstraram elevada disponibilidade de medicamentos anti-hipertensivos no país, com acesso próximo a 98% para tratamento da hipertensão arterial após a expansão das políticas públicas farmacêuticas. A ampliação do acesso a medicamentos para esse grupo de condições crônicas, ofertado pelo Programa Farmácia Popular, também pode ter contribuído para os padrões observados neste estudo.^34^

Além disso, o cuidado multiprofissional na atenção primária à saúde, envolvendo enfermagem, farmácia, educadores físicos, nutrição e agentes comunitários de saúde, pode favorecer maior adesão terapêutica, controle pressórico e redução das complicações cardiovasculares, particularmente em territórios com redes assistenciais mais estruturadas.^35^

Embora a mortalidade prematura por DCV tenha diminuído no Brasil entre 2010 e 2023, as reduções ocorreram de forma desigual entre as regiões. As menores quedas observadas no Norte e em parte do Nordeste indicam que o risco de morrer precocemente por DCV depende não apenas da ampliação do acesso assistencial, mas também da implementação de políticas públicas intersetoriais mais amplas voltadas à redução das desigualdades sociais, promoção da alimentação saudável, melhoria das condições urbanas, segurança alimentar e fortalecimento da atenção primária à saúde.^19,23,28^

Outro aspecto relevante para interpretação das desigualdades regionais observadas refere-se à heterogeneidade histórica da qualidade da informação em mortalidade no Brasil. Regiões como Norte e Nordeste apresentaram, ao longo das últimas décadas, maiores proporções de subregistro de óbitos, causas mal definidas e menor completude das declarações de óbito.^27,28^ Dessa forma, parte das tendências menos favoráveis observadas em alguns estados pode refletir não apenas mudanças reais na mortalidade cardiovascular, mas também avanços graduais na cobertura e qualificação do Sistema de Informações sobre Mortalidade (SIM), incluindo melhorias na codificação das causas básicas de morte e maior capacidade de vigilância epidemiológica nas notificações dos agravos.

As limitações deste estudo devem ser consideradas na interpretação dos resultados, pois foram utilizados dados secundários provenientes do SIM, sujeitos a possíveis inconsistências relacionadas ao preenchimento, subregistro e variabilidade regional da qualidade da informação. Adicionalmente, o período da pandemia de COVID-19 pode ter influenciado a classificação das causas básicas de morte e a organização dos serviços de saúde, impactando parcialmente nas tendências observadas. A ausência de variáveis clínicas individuais e informações mais detalhadas sobre acesso aos serviços de saúde também limita interpretações causais mais robustas. Apesar dessas limitações, o estudo utiliza uma das principais bases nacionais de mortalidade do país, permitindo avaliação abrangente das tendências temporais das DCV em diferentes contextos territoriais brasileiros.

## Conclusões

A análise da mortalidade por doenças cardiovasculares no Brasil evidencia que, embora tenham ocorrido avanços na prevenção, no diagnóstico e no tratamento desse grupo de doenças nas últimas décadas, os benefícios não foram distribuídos de forma equitativa entre os diferentes grupos populacionais e territórios. Persistem importantes desigualdades regionais e socioeconômicas, com maiores taxas de mortalidade concentradas em populações mais vulneráveis, caracterizadas por menor escolaridade, menor renda, piores condições de moradia e maior dificuldade de acesso oportuno aos serviços de saúde. Assim, enfrentar a carga das DCV no país exige não apenas expansão do acesso ao cuidado cardiovascular, mas também fortalecimento de macro políticas voltadas à equidade em saúde e aos determinantes sociais das doenças cardiovasculares. Recomenda-se que pesquisas futuras foquem suas investigações a partir de uma perspectiva interseccional (gênero, raça/cor, classe e território), a fim de identificar subgrupos de maior risco e subsidiar políticas de proteção social e acesso equitativo aos serviços de saúde cardiovascular.

## Material Suplementar

Material Suplementar
