# Supplementary material for: Tendência Temporal da Mortalidade por Doenças Cardiovasculares: Estudo Ecológico das Regiões e Unidades da Federação do Brasil, 2010-2023
Source: Arq Bras Cardiol. 2026 Sep 10;123(8):e20260087. [Article in Portuguese] doi: 10.36660/abc.20260087 (PMC13671139; doi:10.36660/abc.20260087)
Supplement: Supplementary file 2 [file 0066-782x-abc-123-8-e20260087-suppl01.pdf]

Material Suplementar

Tabela S1. Percentual de variação anual (APC) e Percentual de variação anual médio (AAPC) das taxas de mortalidade por Doenças Cardiovasculares padronizadas por idade (por 100 mil habitantes), segundo sexo – Brasil (2010-2023).

UF/Região	Feminino			Masculino		
	Período	APC (IC 95%) p valor	AAPC (IC 95%) p valor	Período	APC (IC 95%) p valor	AAPC (IC 95%) p valor
Acre	2010-2018	1,30 (-6,9 a 8,55); p=0,22	-0,32 (-2,11 a 0,91); p=0,58	2010-2023	-0,81 (-2,37 a 0,7); p=0,29	-0,81 (-2,37 a 0,7); p=0,29
	2018-2021	-8,85 (-14,09 a 8,24); p=0,23				
	2021-2023	6,87 (-6,52 a 17,84); p=0,32				
Alagoas	2010-2023	-0,77 (-1,61 a 0,05); p=0,07	-0,77 (-1,61 a 0,05); p=0,07	2010-2017	0,42 (-3,59 a 4,18); p=0,3	-0,30 (-1,10 a 0,43); p=0,33
				2017-2020	-4,19 (-6,79 a 3,16); p=0,13	
				2020-2023	2,00 (-2,42 a 8,27); p=0,25	
Amapá	2010-2023	2,05* (0,23 a 3,87); p=0,03	2,05* (0,23 a 3,87); p=0,03	2010-2023	1,54* (0,43 a 2,68); p<0,01	1,54* (0,43 a 2,68); p<0,01
Amazonas	2010-2023	0,18 (-1,01 a 1,33); p=0,72	0,18 (-1,01 a 1,33); p=0,72	2010-2015	1,16 (-0,34 a 5,17); p=0,08	1,04* (0,57 a 1,66); p<0,01
				2015-2018	-2,44 (-4,32 a 0,65); p=0,08	
				2018-2023	3,06* (1,39 a 7,42); p=0,02	

Bahia	2010-2019<br>2019-2023	-1,11* (-2,37 a -0,61); p<0,01<br>2,03* (0,3 a 5,56); p=0,02	-0,15 (-0,58 a 0,17); p=0,34	2010-2019<br>2019-2023	-0,84* (-2,06 a -0,41); p<0,01<br>1,50* (0,09 a 4,44); p=0,03	-0,13 (-0,52 a 0,21); p=0,47
Ceará	2017-2023	-2,76* (-6,69 a -1,8); p<0,01	-1,62* (-2,26 a -1); p<0,001	2010-2023	-0,70 (-1,50 a 0,08); p=0,08	-0,70 (-1,50 a 0,08); p=0,08
Distrito Federal	2010-2023	-4,15* (-4,59 a -3,7); p<0,001	-4,15* (-4,59 a -3,7); p<0,001	2010-2023	-3,58* (-4,25 a -2,93); p<0,001	-3,58* (-4,25 a -2,93); p<0,001
Espírito Santo	2010-2013<br>2013-2023	-4,93* (-7,96 a -2,55); p<0,001<br>-2,29 (-3,61 a 0,15); p=0,06	-2,9* (-3,27 a -2,35); p<0,001	2010-2012<br>2012-2023	-6,19* (-8,81 a -2,23); p<0,001<br>-1,87 (-2,39 a 0); p=0,05	-2,54* (-2,93 a -1,94); p<0,001
Goiás	2010-2012<br>2012-2016<br>2016-2023	3,80 (-0,66 a 8,61); p=0,1<br>-5,02* (-7,50 a -3,06); p<0,01<br>-0,92 (-1,87 a 1,89); p=0,32	-1,5* (-1,98 a -0,87); p<0,001	2010-2023	-1,29* (-1,91 a -0,68); p<0,001	-1,29* (-1,91 a -0,68); p<0,001
Maranhão	2010-2015<br>2015-2023	1,36 (-0,1 a 5,93); p=0,07<br>-1,47* (-4,5 a -0,76); p<0,01	-0,39 (-1,05 a 0,26); p=0,19	2010-2023	-0,31 (-0,9 a 0,26); p=0,29	-0,31 (-0,90 a 0,26); p=0,29
Mato Grosso	2010-2020<br>2020-2023	-2,70* (-3,17 a -2,39); p<0,001<br>1,05 (-0,76 a 3,71); p=0,23	-1,84* (-2,16 a -1,6); p<0,001	2010-2012<br>2012-2020<br>2020-2023	-8,02* (-10,34 a -3,56); p<0,001<br>-1,78 (-3,33 a 0,03); p=0,05<br>1,18 (-1,33 a 4,48); p=0,35	-2,1* (-2,59 a -1,51); p<0,001
Mato Grosso do Sul	2010-2023	-1,37* (-2,52 a -0,26); p=0,02	-1,37* (-2,52 a -0,26); p=0,02	2010-2017	-2,75* (-7,05 a -1,44); p=0,02	-1,37* (-2,06 a -0,72); p<0,001

				2017-2023	0,26 (-1,36 a 5,47); p=0,66	
Minas Gerais	2010-2019	-2,75* (-4,71 a -2,11); p<0,01	-2,24* (-2,64 a -1,9); p<0,001	2010-2018	-2,74* (-4,19 a -2,28); p<0,001	-1,97* (-2,30 a -1,69); p<0,001
	2019-2023	-1,09 (-2,42 a 1,69); p=0,35		2018-2023	-0,73 (-1,72 a 1,85); p=0,35	
Paraná	2010-2023	-2,77* (-3,33 a -2,20); p<0,001	-2,77* (-3,33 a -2,2); p<0,001	2010-2020	-2,81* (-5,53 a -1,89); p=0,03	-2,07* (-2,72 a -1,56); p<0,001
				2020-2023	0,42 (-2,43 a 4,53); p=0,87	
Paraíba	2010-2023	-2,46* (-3,2 a -1,74); p<0,001	-2,46* (-3,2 a -1,74); p<0,001	2010-2023	-1,73* (-2,34 a -1,11); p<0,001	-1,73* (-2,34 a -1,11); p<0,001
Pará	2010-2015	1,10 (-0,14 a 5,34); p=0,1	-0,37 (-1,02 a 0,26); p=0,15	2010-2015	0,88* (0,09 a 3,06); p=0,02	-0,16 (-0,47 a 0,19); p=0,23
	2015-2023	-1,29* (-4,3 a -0,67); p<0,01		2015-2023	-0,80* (-1,93 a -0,41); p<0,01	
Pernambuco	2010-2023	-2,61* (-3,3 a -1,94); p<0,001	-2,61* (-3,3 a -1,94); p<0,001	2010-2017	-1,25 (-2,13 a 1,47); p=0,15	-1,64* (-2,35 a -1,05); p<0,001
				2017-2021	-5,2* (-8,31 a -3,11); p=0,02	
				2021-2023	4,43 (-1,68 a 9,11); p=0,12	
Piauí	2010-2023	-1,86* (-2,7 a -1,04); p<0,001	-1,86* (-2,70 a -1,04); p<0,001	2010-2023	-1,33* (-1,98 a -0,7); p<0,001	-1,33* (-1,98 a -0,7); p<0,001
Rio Grande do Norte	2010-2023	-1,07* (-1,77 a -0,37); p<0,01	-1,07* (-1,77 a -0,37); p<0,01	2010-2023	-0,42 (-1,22 a 0,38); p=0,29	-0,42 (-1,22 a 0,38); p=0,29
Rio Grande do Sul	2010-2023	-3,15* (-3,86 a -2,42); p<0,001	-3,15* (-3,86 a -2,42); p<0,001	2010-2020	-3,36* (-6,30 a -2,64); p=0,01	-2,51* (-3,22 a -1,94); p<0,001
				2020-2023	0,37 (-2,70 a 5,26); p=0,89	
Rio de Janeiro	2010-2023	-2,34* (-2,96 a -1,75); p<0,001	-2,34* (-2,96 a -1,75); p<0,001	2010-2023	-2,37* (-2,76 a -1,98); p<0,001	-2,37* (-2,76 a -1,98); p<0,001

Rondônia	2010-2017	-0,94 (-1,88 a 0,93); p=0,17	-1,68* (-2,23 a -1,17); p<0,001	2010-2020	-2,26* (-4,63 a -1,57); p=0,01	-1,16* (-1,89 a -0,66); p<0,001
	2017-2020	-5,54* (-7,38 a -1,86); p=0,04		2020-2023	2,62 (-1,07 a 7,56); p=0,23	
	2020-2023	0,57 (-2,76 a 5,16); p=0,54				
Roraima	2010-2023	0,63 (-1,41 a 2,62); p=0,49	0,63 (-1,41 a 2,62); p=0,49	2010-2023	0,66 (-1,27 a 2,54); p=0,43	0,66 (-1,27 a 2,54); p=0,43
Santa Catarina	2010-2020	-3,03* (-6,83 a -0,53); p=0,05	-2,19* (-3 a -1,57); p<0,001	2010-2019	-2,95 (-7,84 a 3,04); p=0,07	-1,98* (-2,92 a -1,21); p<0,001
	2020-2023	0,66 (-2,82 a 5,76); p=0,85		2019-2023	0,24 (-5,01 a 6,29); p=0,94	
São Paulo	2010-2023	-1,86* (-2,38 a -1,34); p<0,001	-1,86* (-2,38 a -1,34); p<0,001	2010-2020	-1,94* (-4,17 a -1,14); p=0,03	-1,3* (-1,86 a -0,85); p<0,001
				2020-2023	0,88 (-1,58 a 4,52); p=0,61	
Sergipe	2010-2023	-2,8* (-3,68 a -1,95); p<0,001	-2,8* (-3,68 a -1,95); p<0,001	2010-2017	-3,2* (-6,42 a -2,33); p<0,01	-2,08* (-2,65 a -1,52); p<0,001
				2017-2023	-0,75 (-1,88 a 3,06); p=0,52	
Tocantins	2010-2023	-2,29* (-2,88 a -1,71); p<0,001	-2,29* (-2,88 a -1,71); p<0,001	2010-2012	-7,2* (-11,86 a -0,83); p=0,01	-1,79* (-2,51 a -0,73); p<0,01
				2012-2023	-0,77 (-3,71 a 3,49); p=0,46	
Região Norte	2010-2023	-0,7* (-1,04 a -0,36); p<0,001	-0,7* (-1,04 a -0,36); p<0,001	2010-2015	0,88* (0,22 a 2,43); p=0,03	0,05 (-0,2 a 0,34); p=0,45
				2015-2018	-1,63* (-2,53 a -0,27); p=0,04	
				2018-2023	0,23 (-0,64 a 2,1); p=0,31	
Região Nordeste	2010-2023	-1,46* (-1,89 a -1,03); p<0,001	-1,46* (-1,89 a -1,03); p<0,001	2010-2023	-0,87* (-1,38 a -0,38); p<0,01	-0,87* (-1,38 a -0,38); p<0,01

Região Centro-Oeste	2010-2019<br>2019-2023	-2,81 (-5,79 a 0,31); p=0,05<br>-0,73 (-2,69 a 2,91); p=0,51	-2,18* (-2,73 a -1,73); p<0,001	2010-2019<br>2019-2023	-2,55* (-2,95 a -2,27); p<0,001<br>0,43 (-0,55 a 2,48); p=0,26	-1,64* (-1,86 a -1,47); p<0,001
Região Sudeste	2010-2023	-2,08* (-2,49 a -1,67); p<0,001	-2,08* (-2,49 a -1,67); p<0,001	2010-2020<br>2020-2023	-2,28* (-3,84 a -1,88); p<0,01<br>0,27 (-1,68 a 3,22); p=0,85	-1,7* (-2,13 a -1,35); p<0,001
Região Sul	2010-2020<br>2020-2023	-3,45* (-6,2 a -2,74); p<0,01<br>0,18 (-2,79 a 4,95); p=0,97	-2,63* (-3,33 a -2,06); p<0,001	2010-2020<br>2020-2023	-3,06* (-6,82 a -0,3); p=0,05<br>0,6 (-2,87 a 5,63); p=0,86	-2,23* (-3,05 a -1,62); p<0,001
Brasil	2010-2023	-2,01* (-2,36 a -1,65); p<0,001	-2,01* (-2,36 a -1,65); p<0,001	2010-2020<br>2020-2023	-1,95* (-3,56 a -1,51); p=0,01<br>-0,07 (-1,66 a 2,4); p=0,89	-1,52* (-1,9 a -1,21); p<0,001

UF: Unidade da Federação; IC 95%: Intervalo de Confiança de 95%; *estatisticamente significativo

Tabela S2 – Percentual de variação anual (APC) e Percentual de variação anual médio (AAPC) das taxas de mortalidade por Doenças Cerebrovasculares padronizadas por idade (por 100 mil habitantes), segundo sexo – Brasil (2010-2023).

**UF/Região**	**Feminino**			**Masculino**		
	**Período**	**APC (IC 95%) p valor**	**AAPC (IC 95%) p valor**	**Período**	**APC (IC 95%) p valor**	**AAPC (IC 95%) p valor**
Acre	2010-2023	-3,1* (-5,2 a -1); p<0,01	-3,1* (-5,2 a -1,02); p<0,01	2010-2013<br>2013-2023	6,67 (-2,28 a 31,94); p=0,2<br>-4,13* (-20,51 a -2,2); p=0,02	-1,74 (-4,9 a 0,91); p=0,12
Alagoas	2010-2019	-1,36 (-2,61 a 6,29); p=0,26	-2,5* (-3,67 a -1,17); p<0,001	2010-2023	-2,23* (-3,14 a -1,35); p<0,001	-2,23* (-3,14 a -1,35); p<0,001

	2019-2023	-5,01* (-13,46 a -1,98); p<0,01				
Amapá	2010-2023	0,67 (-1,17 a 2,46); p=0,42	0,67 (-1,17 a 2,46); p=0,42	2010-2023	0,76 (-0,92 a 2,42); p=0,34	0,76 (-0,92 a 2,42); p=0,34
Amazonas	2010-2014	4,17* (1,02 a 12,19); p<0,01	0,32 (-0,64 a 1,55); p=0,35	2010-2023	-0,05 (-1,2 a 1,09); p=0,95	-0,05 (-1,2 a 1,09); p=0,95
	2014-2019	-4,28* (-9,64 a -2,02); p<0,01				
	2019-2023	2,45 (-1,3 a 10,76); p=0,14				
Bahia	2010-2020	-2,2* (-3,39 a -1,71); p<0,001	-1,2* (-1,75 a -0,72); p<0,001	2010-2021	-1,31 (-4,73 a 2,75); p=0,07	-0,45 (-1,34 a 0,06); p=0,08
	2020-2023	2,21 (-0,63 a 6,6); p=0,13		2021-2023	4,43 (-1,28 a 8,09); p=0,25	
Ceará	2010-2017	-2,03 (-3,12 a 3,65); p=0,15	-3,42* (-4,44 a -2,41); p<0,001	2010-2016	-0,89 (-2,69 a 8,25); p=0,56	-2,63* (-3,98 a -1,3); p<0,001
	2017-2023	-5,03* (-11,7 a -3,67); p<0,001		2016-2023	-4,09* (-12,54 a -2,64); p<0,01	
Distrito Federal	2010-2017	-2,45 (-4,61 a 11); p=0,26	-4,55* (-6,44 a -2,58); p<0,001	2010-2017	-0,7 (-2,42 a 7,86); p=0,66	-2,89* (-4,31 a -1,4); p<0,001
	2017-2023	-6,93* (-19,08 a -4,35); p<0,01		2017-2023	-5,38* (-14 a -3,26); p<0,001	
Espírito Santo	2010-2023	-4,17* (-4,88 a -3,48); p<0,001	-4,17* (-4,88 a -3,48); p<0,001	2010-2023	-4,07* (-4,82 a -3,34); p<0,001	-4,07* (-4,82 a -3,34); p<0,001
Goiás	2010-2018	-2,98* (-6,99 a -1,71); p=0,04	-1,95* (-2,64 a -1,36); p<0,001	2010-2018	-2,57* (-5,8 a -1,64); p=0,03	-1,71* (-2,27 a -1,21); p<0,001
	2018-2023	-0,28 (-2,25 a 4,72); p=0,85		2018-2023	-0,33 (-1,84 a 3,61); p=0,82	
Maranhão	2010-2023	-2,18* (-3,15 a -1,24); p<0,001	-2,18* (-3,15 a -1,24); p<0,001	2010-2023	-1,93* (-2,72 a -1,17); p<0,001	-1,93* (-2,72 a -1,17); p<0,001
Mato Grosso	2010-2023	-3,41* (-4,25 a -2,57); p<0,001	-3,41* (-4,25 a -2,57); p<0,001	2010-2012	-9,35* (-11,94 a -3,7); p<0,001	-3,87* (-4,3 a -3,07); p<0,001

				2012-2023	-2,84* (-3,46 a -0,9); p=0,03	
Mato Grosso do Sul	2010-2023	-3,06* (-4,27 a -1,87); p<0,001	-3,06* (-4,27 a -1,87); p<0,001	2010-2021	-3,18 (-8,2 a 0,7); p=0,05	-1,95* (-3,3 a -1,09); p<0,001
				2021-2023	5,07 (-3,2 a 11,67); p=0,47	
Minas Gerais	2010-2020	-3,97* (-6,21 a -3,34); p<0,01	-3,31* (-3,85 a -2,92); p<0,001	2010-2012	-7,05* (-8,37 a -4,62); p<0,001	-3,28* (-3,59 a -2,97); p<0,001
	2020-2023	-1,07 (-3,55 a 2,28); p=0,46		2012-2021	-3,06* (-3,74 a -2,38); p<0,001	
				2021-2023	-0,37 (-2,74 a 0,99); p=0,42	
Paraná	2010-2023	-3,47* (-4,26 a -2,7); p<0,001	-3,47* (-4,26 a -2,7); p<0,001	2010-2020	-3,7* (-5,45 a -3,11); p<0,001	-2,62* (-3,3 a -2,02); p<0,001
				2020-2023	1,07 (-2,29 a 6,26); p=0,58	
Paraíba	2010-2023	-4,54* (-5,4 a -3,69); p<0,001	-4,54* (-5,4 a -3,69); p<0,001	2010-2023	-3,41* (-4,31 a -2,54); p<0,001	-3,41* (-4,31 a -2,54); p<0,001
Pará	2010-2016	0,26 (-1,02 a 3,11); p=0,64	-1,9* (-2,51 a -1,24); p<0,001	2010-2015	0,78 (-0,6 a 3,6); p=0,24	-1,59* (-2,06 a -1,06); p<0,001
	2016-2023	-3,71* (-5,6 a -2,7); p<0,001		2015-2023	-3,04* (-4,2 a -2,34); p<0,001	
Pernambuco	2010-2023	-3,35* (-4,29 a -2,41); p<0,001	-3,35* (-4,29 a -2,41); p<0,001	2010-2023	-2,65* (-3,2 a -2,12); p<0,001	-2,65* (-3,2 a -2,12); p<0,001
Piauí	2010-2023	-3,09* (-3,69 a -2,5); p<0,001	-3,09* (-3,69 a -2,5); p<0,001	2010-2023	-2,36* (-3,29 a -1,45); p<0,001	-2,36* (-3,29 a -1,45); p<0,001
Rio Grande do Norte	2010-2023	-2,7* (-3,45 a -1,98); p<0,001	-2,7* (-3,45 a -1,98); p<0,001	2010-2023	-2,33* (-3,3 a -1,33); p<0,001	-2,33* (-3,3 a -1,33); p<0,001
Rio Grande do Sul	2010-2023	-4,26* (-5,1 a -3,38); p<0,001	-4,26* (-5,1 a -3,38); p<0,001	2010-2023	-3,61* (-4,43 a -2,8); p<0,001	-3,61* (-4,43 a -2,8); p<0,001
Rio de Janeiro	2010-2019	-3,68* (-7,28 a -1,12); p=0,04	-2,87* (-3,56 a -2,32); p<0,001	2010-2015	-4,91* (-8,43 a -3,38); p<0,001	-2,88* (-3,35 a -2,35); p<0,001
	2019-2023	-1,02 (-3,43 a 3,5); p=0,54		2015-2023	-1,59 (-2,37 a 0,37); p=0,08	

Rondônia	2010-2023	-3,29* (-4,15 a -2,43); p<0,001	-3,29* (-4,15 a -2,43); p<0,001	2010-2023	-1,75* (-3,04 a -0,45); p<0,01	-1,75* (-3,04 a -0,45); p<0,01
Roraima	2010-2023	1,15 (-0,86 a 3,23); p=0,26	1,15 (-0,86 a 3,23); p=0,26	2010-2023	1,57 (-0,96 a 4,13); p=0,21	1,57 (-0,96 a 4,13); p=0,21
Santa Catarina	2010-2023	-3,44* (-4,33 a -2,56); p<0,001	-3,44* (-4,33 a -2,56); p<0,001	2010-2017	-4,82* (-9,39 a -3,49); p<0,01	-3,17* (-3,96 a -2,41); p<0,001
				2017-2023	-1,22 (-2,87 a 4,18); p=0,49	
São Paulo	2010-2023	-3,01* (-3,41 a -2,63); p<0,001	-3,01* (-3,41 a -2,63); p<0,001	2010-2018	-3,31* (-4,55 a -2,93); p<0,001	-2,75* (-3,04 a -2,45); p<0,001
				2018-2023	-1,85 (-2,6 a 0,17); p=0,06	
Sergipe	2010-2023	-3,7* (-4,8 a -2,6); p<0,001	-3,74* (-4,81 a -2,65); p<0,001	2010-2014	-1,4 (-3,98 a 4,65); p=0,46	-2,54* (-3,16 a -1,66); p<0,001
				2014-2017	-7,27* (-9,58 a -2,74); p=0,02	
				2017-2023	-0,85 (-2,78 a 4,56); p=0,57	
Tocantins	2010-2023	-3,03* (-4,14 a -1,92); p<0,001	-3,03* (-4,14 a -1,92); p<0,001	2010-2020	-3,33* (-6,11 a -2,51); p<0,01	-1,94* (-2,85 a -1,34); p<0,001
				2020-2023	2,83 (-1,72 a 8,52); p=0,26	
Região Norte	2010-2015	-0,32 (-1,45 a 3,11); p=0,68	-1,7* (-2,23 a -1,15); p<0,001	2010-2015	0,33 (-0,18 a 1,13); p=0,17	-1,1* (-1,26 a -0,89); p<0,001
	2015-2023	-2,55* (-4,64 a -2); p<0,001		2015-2018	-3,56* (-4,27 a -2,32); p<0,001	
				2018-2023	-1,02 (-1,53 a 0,18); p=0,09	
Região Nordeste	2010-2023	-2,78* (-3,3 a -2,27); p<0,001	-2,78* (-3,3 a -2,27); p<0,001	2010-2023	-2,14* (-2,71 a -1,58); p<0,001	-2,14* (-2,71 a -1,58); p<0,001
Região Centro-Oeste	2010-2017	-2,43* (-2,73 a -1,58); p<0,01	-2,59* (-2,8 a -2,36); p<0,001	2010-2021	-2,7* (-4,53 a -1,12); p=0,03	-2,23* (-2,72 a -1,94); p<0,001

	2017-2020	-4,69* (-5,51 a -3,47); p<0,001		2021-2023	0,39 (-2,68 a 2,41); p=0,89	
	2020-2023	-0,82 (-2,05 a 1,3); p=0,32				
Região Sudeste	2010-2020	-3,39* (-4,68 a -3,04); p<0,001	-2,86* (-3,23 a -2,59); p<0,001	2010-2018	-3,61* (-5,57 a -3,08); p<0,001	-2,86* (-3,23 a -2,55); p<0,001
	2020-2023	-1,07 (-2,84 a 1,38); p=0,31		2018-2023	-1,66 (-2,72 a 1,14); p=0,16	
Região Sul	2010-2023	-3,78* (-4,39 a -3,16); p<0,001	-3,78* (-4,39 a -3,16); p<0,001	2010-2020	-3,99* (-7,28 a -3,11); p=0,02	-2,98* (-3,8 a -2,35); p<0,001
				2020-2023	0,47 (-3,3 a 5,71); p=0,89	
Brasil	2010-2016	-2,74* (-3,24 a -0,26); p=0,04	-2,71* (-3,03 a -2,35); p<0,001	2010-2020	-3,03* (-3,69 a -2,76); p<0,001	-2,48* (-2,79 a -2,22); p<0,001
	2016-2020	-4,19* (-5,79 a -3,3); p<0,001				
	2020-2023	-0,62 (-2,51 a 2,12); p=0,48		2020-2023	-0,6 (-2,21 a 1,75); p=0,45	

UF: Unidade da Federação; IC 95%: Intervalo de Confiança de 95%; *estatisticamente significativo

Tabela S3.Percentual de variação anual (APC) e Percentual de variação anual médio (AAPC) das taxas de mortalidade por Doenças Hipertensivas padronizadas por idade (por 100 mil habitantes), segundo sexo – Brasil (2010-2023).

**UF/Região**	**Feminino**			**Masculino**		
	**Período**	**APC (IC 95%) p valor**	**AAPC (IC 95%) p valor**	**Período**	**APC (IC 95%) p valor**	**AAPC (IC 95%) p valor**
Acre	2010-2018	2,79 (-1,87 a 22,88); p=0,23	-4,4* (-7,89 a -0,4); p=0,04	2010-2023	-2,44 (-6,39 a 1,6); p=0,22	-2,44 (-6,39 a 1,6); p=0,22

	2018-2023	-14,87* (-33,85 a -6,29); p<0,001				
Alagoas	2010-2023	-0,15 (-1,39 a 1,07); p=0,82	-0,15 (-1,39 a 1,07); p=0,82	2010-2023	-0,17 (-1,31 a 0,96); p=0,75	-0,17 (-1,31 a 0,96); p=0,75
Amapá	2010-2023	3,92* (0,2 a 7,62); p=0,04	3,92* (0,2 a 7,62); p=0,04	2010-2023	2,36 (-0,48 a 5,25); p=0,1	2,36 (-0,48 a 5,25); p=0,1
Amazonas		2,26* (0,68 a 3,87); p<0,01	2,26* (0,68 a 3,87); p<0,01	2010-2023	3,28* (1,29 a 5,23); p<0,01	3,28* (1,29 a 5,23); p<0,01
Bahia	2010-2016	-1,61 (-11,37 a 1,93); p=0,24	1,5* (0,1 a 2,97); p=0,04	2010-2016	-1,3 (-9,2 a 1,2); p=0,29	1,38* (0,18 a 2,62); p=0,03
	2016-2023	4,23* (1,15 a 14,93); p=0,04		2016-2023	3,73* (1,61 a 11,92); p=0,01	
Ceará	2010-2018	-5,21 (-15,66 a 6,14); p=0,07	-2,77* (-4,68 a -1,08); p<0,01	2010-2018	-5,88* (-17,94 a -0,42); p=0,05	-2,72* (-4,95 a -0,76); p<0,01
	2018-2023	1,27 (-7 a 15,66); p=0,57		2018-2023	2,56 (-4,09 a 20,42); p=0,4	
Distrito Federal	2010-2020	0,4 (-1,79 a 11,87); p=0,49	-1,89 (-4,02 a 0,58); p=0,09	2010-2015	-3,23 (-13,05 a 0,61); p=0,09	-1,89* (-3,54 a -0,55); p=0,01
	2020-2023	-9,17* (-23,73 a -0,86); p=0,03		2015-2018	11,56* (2,59 a 17,78); p=0,01	
				2018-2023	-7,9* (-15,92 a -4,29); p<0,01	
Espírito Santo	2010-2018	-1,15 (-5,68 a 0,25); p=0,1	0,77 (-0,22 a 1,7); p=0,15	2010-2018	-1,78 (-9,6 a 0,9); p=0,1	0,33 (-1,08 a 1,62); p=0,67
	2018-2023	3,92* (1,17 a 11,31); p<0,01		2018-2023	3,81 (-0,17 a 14,7); p=0,06	
Goiás	2010-2023	-0,01 (-1,7 a 1,69); p=0,99	-0,01 (-1,7 a 1,69); p=0,99	2010-2023	0,49 (-1,19 a 2,15); p=0,51	0,49 (-1,19 a 2,15); p=0,51
Maranhão	2010-2023	0,24 (-1,08 a 1,53); p=0,69	0,24 (-1,08 a 1,53); p=0,69	2010-2023	-0,09 (-1,11 a 0,95); p=0,86	-0,09 (-1,11 a 0,95); p=0,86
Mato Grosso	2010-2018	-3,18* (-4,4 a -2,27); p<0,001	-0,74* (-1,28 a -0,19); p=0,01	2010-2014	-8,13* (-15,75 a -4,43); p<0,001	-1,24* (-1,95 a -0,32); p=0,01

	2018-2023	3,28* (1,43 a 6,41); p<0,001		2014-2023	1,99* (0,8 a 3,91); p<0,001	
Mato Grosso do Sul	2010-2023	-1,72* (-3,03 a -0,42); p<0,01	-1,72* (-3,03 a -0,42); p<0,01	2010-2023	-2,41* (-3,94 a -0,89); p<0,01	-2,41* (-3,94 a -0,89); p<0,01
Minas Gerais	2010-2015	-2,76* (-6,48 a -0,73); p<0,01	0,09 (-0,64 a 0,75); p=0,72	2010-2015	-2,23 (-8,9 a 0,51); p=0,12	1,02* (0,17 a 2,02); p=0,02
	2015-2021	3,96* (2,56 a 8,01); p<0,01		2015-2023	3,11* (1,74 a 8,04); p<0,01	
	2021-2023	-3,97 (-8,71 a 0,89); p=0,13				
Paraná	2010-2014	-3,57 (-11,9 a 1,08); p=0,14	0,34 (-0,72 a 1,7); p=0,49	2010-2023	1,39* (0,32 a 2,45); p<0,01	1,39* (0,32 a 2,45); p<0,01
	2014-2023	2,13 (-0,6 a 9,29); p=0,06				
Paraíba	2010-2018	-2,75 (-11,52 a 0,08); p=0,05	-0,4 (-1,98 a 1,01); p=0,54	2010-2018	-3,83 (-8 a 0,53); p=0,05	-1,6* (-3,01 a -0,12); p=0,04
	2018-2023	3,49 (-1,12 a 15,86); p=0,12		2018-2021	10,76 (-5,63 a 15,92); p=0,11	
				2021-2023	-9,68 (-18,44 a 3,5); p=0,12	
Pará	2010-2023	0,81 (-0,44 a 2,07); p=0,18	0,81 (-0,44 a 2,07); p=0,18	2010-2023	0,63 (-0,54 a 1,8); p=0,29	0,63 (-0,54 a 1,8); p=0,29
Pernambuco	2010-2018	-2,2 (-9,14 a 0,39); p=0,08	-0,98 (-3,08 a 0,52); p=0,16	2010-2018	-2,22 (-10,05 a 1); p=0,12	-0,39 (-2,74 a 1,49); p=0,62
	2018-2021	15,58* (4,4 a 22,59); p=0,02		2018-2021	17,88* (3,33 a 26,23); p=0,04	
	2021-2023	-17,48* (-28,86 a -3,61); p=0,03		2021-2023	-16,63 (-29,32 a 1,63); p=0,06	
Piauí	2010-2023	-1,54* (-2,75 a -0,37); p=0,01	-1,54* (-2,75 a -0,37); p=0,01	2010-2023	-0,99* (-1,83 a -0,15); p=0,02	-0,39 (-2,74 a 1,49); p=0,62
Rio Grande do Norte	2010-2015	2,96 (-1,37 a 20,65); p=0,18	-0,92 (-3,29 a 1,31); p=0,29	2010-2023	-0,81 (-1,73 a 0,08); p=0,07	-0,81 (-1,73 a 0,08); p=0,07

	2015-2023	-3,27* (-16,62 a -0,83); p=0,03				
Rio Grande do Sul	2010-2023	1,86* (0,49 a 3,23); p<0,01	1,86* (0,49 a 3,23); p<0,01	2010-2023	1,93* (0,74 a 3,1); p<0,001	1,93* (0,74 a 3,1); p<0,001
Rio de Janeiro	2010-2015	-6,59* (-9,15 a -4,72); p<0,001	-1,49* (-2,15 a -0,88); p<0,001	2010-2015	-6,37* (-8,95 a -4,41); p<0,001	-1,28* (-1,94 a -0,56); p<0,001
	2015-2021	4,18* (2,98 a 7,76); p=0,01		2015-2021	4,75* (3,33 a 8,43); p<0,01	
				2021-2023	-5,65 (-9,89 a 0,01); p=0,05	
Rondônia	2010-2023	-1,67* (-2,88 a -0,43); p<0,01	-1,67* (-2,88 a -0,43); p<0,01	2010-2020	-2,68* (-6,5 a -1,15); p<0,01	0,39 (-1,33 a 1,45); p=0,53
				2020-2023	11,33* (1,75 a 24,02); p<0,01	
Roraima	2010-2012	27,22 (-6,21 a 79,74); p=0,21	-1,12 (-7,1 a 3,61); p=0,36	2010-2023	-3,76* (-6,59 a -0,98); p=0,01	-3,76* (-6,59 a -0,98); p=0,01
	2012-2023	-5,55 (-29,79 a 2,74); p=0,08				
Santa Catarina	2010-2023	0,03 (-1,7 a 1,73); p=0,95	0,03 (-1,7 a 1,73); p=0,95	2010-2023	0,25 (-1,09 a 1,56); p=0,67	0,25 (-1,09 a 1,56); p=0,67
São Paulo	2010-2018	-1,94* (-4,57 a -0,7); p<0,01	-1,22* (-2,26 a -0,46); p<0,01	2010-2018	-1,09 (-7,93 a 0,88); p=0,15	-0,95 (-2,55 a 0,16); p=0,08
	2018-2021	9,63* (3,88 a 13,06); p<0,01		2018-2021	9,13* (2,17 a 13,92); p=0,02	
	2021-2023	-12,99* (-19,26 a -5,14); p<0,01		2021-2023	-13,89* (-22,99 a -3,51); p=0,02	
Sergipe	2010-2023	-3,53* (-4,93 a -2,11); p<0,001	-3,53* (-4,93 a -2,11); p<0,001	2010-2023	-2,99* (-4,75 a -1,24); p<0,001	-2,99* (-4,75 a -1,24); p<0,001
Tocantins	2010-2023	-1,71* (-3,26 a -0,15); p=0,03	-1,71* (-3,26 a -0,15); p=0,03			-1,11 (-3,54 a 1,27); p=0,34
Região Norte	2010-2023	0,06 (-1 a 1,12); p=0,88	0,06 (-1 a 1,12); p=0,88	2010-2023	0,42 (-0,37 a 1,19); p=0,27	0,42 (-0,37 a 1,19); p=0,27

Região Nordeste	2010-2018	-2,04 (-7,55 a 5,5); p=0,11	-0,87 (-2,17 a 0,77); p=0,12	2010-2018	-2,08 (-5,51 a 0,35); p=0,05	-0,82 (-1,89 a 0,31); p=0,08
	2018-2021	6,03 (-4,87 a 9,66); p=0,2		2018-2021	7,37 (-3,1 a 10,92); p=0,09	
	2021-2023	-6,04 (-13,95 a 3,48); p=0,24		2021-2023	-7,31 (-14,06 a 2,26); p=0,11	
Região Centro-Oeste	2010-2023	-0,81 (-5,58 a 4,29); p=0,76	-0,81 (-5,58 a 4,29); p=0,76	2010-2017	5,69* (0,83 a 28,68); p=0,02	-0,25 (-3,59 a 3,24); p=0,89
				2017-2023	-6,75* (-25,75 a -1,01); p=0,02	
Região Sudeste	2010-2015	-4,49* (-8,06 a -2,25); p<0,01	-1,1* (-1,88 a -0,42); p<0,01	2010-2015	-3,88* (-6,96 a -1,82); p<0,001	-0,66 (-1,36 a 0,02); p=0,06
	2015-2021	3,64* (2,26 a 8,08); p<0,01		2015-2021	4,12* (2,76 a 8,16); p<0,01	
	2021-2023	-6,23* (-11,25 a -0,92); p=0,02		2021-2023	-6,31* (-10,8 a -1,03); p=0,01	
Região Sul	2010-2023	0,34 (-2,26 a 2,98); p=0,79	0,34 (-2,26 a 2,98); p=0,79	2010-2023	1,93 (-1,88 a 5,88); p=0,33	1,93 (-1,88 a 5,88); p=0,33
Brasil	2010-2018	-1,65 (-6,14 a 2,7); p=0,08	-0,63 (-1,72 a 0,55); p=0,12	2010-2018	-1,28 (-4,42 a 0,28); p=0,07	-0,34 (-1,26 a 0,5); p=0,32

UF: Unidade da Federação; IC 95%: Intervalo de Confiança de 95%; *estatisticamente significativo

Tabela S4. Percentual de variação anual (APC) e Percentual de variação anual médio (AAPC) das taxas de mortalidade por Doenças Isquêmicas do Coração padronizadas por idade(por 100 mil habitantes), segundo sexo – Brasil (2010-2023).

**UF/Região**	**Feminino**			**Masculino**		
	**Período**	**APC (IC 95%) p valor**	**AAPC (IC 95%) p valor**	**Período**	**APC (IC 95%) p valor**	**AAPC (IC 95%) p valor**
Acre	2010-2023	2,28 (-0,87 a 5,48); p=0,15	2,28 (-0,87 a 5,48); p=0,15	2010-2023	1,86 (-1,74 a 5,46); p=0,27	1,86 (-1,74 a 5,46); p=0,27

Alagoas	2010-2012	17,97* (3,69 a 29,32); p<0,001	2,62* (0,93 a 3,99); p<0,01	2010-2016	3,29* (1,35 a 10,56); p<0,01	0,92 (-0,22 a 2,05); p=0,1
	2012-2023	0,06 (-2,81 a 1,2); p=0,83		2016-2023	-1,08 (-7,46 a 0,4); p=0,18	
Amapá	2010-2015	17,87 (-1,11 a 121,54); p=0,06	7,19 (-2,23 a 16,63); p=0,1	2010-2015	13,72* (8,4 a 25,7); p<0,001	5,21* (3,44 a 7,14); p<0,001
	2015-2023	1,02 (-45,96 a 17,06); p=0,92		2015-2023	0,21 (-4,07 a 2,85); p=0,97	
Amazonas	2010-2023	-0,46 (-1,71 a 0,79); p=0,46	-0,46 (-1,71 a 0,79); p=0,46	2010-2023	0,24 (-0,68 a 1,15); p=0,55	0,24 (-0,68 a 1,15); p=0,55
Bahia	2010-2023	-0,07 (-0,65 a 0,51); p=0,81	-0,07 (-0,65 a 0,51); p=0,81	2010-2023	0,23 (-0,42 a 0,87); p=0,43	0,23 (-0,42 a 0,87); p=0,43
Ceará	2010-2018	1,66 (-0,03 a 6,81); p=0,06	-1,29 (-2,98 a 0,23); p=0,07	2010-2018	2,36* (1,04 a 7,62); p<0,01	0,35 (-0,8 a 1,58); p=0,48
	2018-2023	-5,83* (-14,66 a -2,49); p<0,001		2018-2023	-2,78* (-10,07 a -0,17); p=0,03	
Distrito Federal	2010-2023	-2* (-3,98 a -0,03); p=0,05	-2* (-3,98 a -0,03); p=0,05	2010-2017	0,76 (-0,55 a 7,83); p=0,25	-0,97 (-2,18 a 0,24); p=0,08
				2017-2023	-2,95* (-10,38 a -1,31); p<0,01	
Espírito Santo	2010-2023	-3* (-3,73 a -2,26); p<0,001	-3* (-3,73 a -2,26); p<0,001	2010-2023	-1,9* (-2,82 a -0,98); p<0,001	-1,9* (-2,82 a -0,98); p<0,001
Goiás	2010-2012	9,46* (0,68 a 19,48); p=0,03	0,08 (-1 a 1,27); p=0,93	2010-2023	-0,55 (-1,12 a 0,05); p=0,07	-0,55 (-1,12 a 0,05); p=0,07
	2012-2020	-3,73* (-9,42 a -2,73); p<0,001				
	2020-2023	4,57 (-0,97 a 12,63); p=0,13				
Maranhão	2010-2015	5,59* (2,67 a 12,08); p<0,001	0,41 (-0,6 a 1,58); p=0,38	2010-2015	4,81* (2,52 a 10); p<0,001	0,65 (-0,15 a 1,66); p=0,1
	2015-2023	-2,7* (-5,18 a -1,2); p<0,001		2015-2023	-1,87* (-3,78 a -0,7); p<0,01	

Mato Grosso	2010-2023	-1,23* (-2,39 a -0,08); p=0,04	-1,23* (-2,39 a -0,08); p=0,04	2010-2023	-1,16* (-2,18 a -0,15); p=0,03	-1,16* (-2,18 a -0,15); p=0,03
Mato Grosso do Sul	2010-2023	-0,59 (-1,7 a 0,53); p=0,28	-0,59 (-1,7 a 0,53); p=0,28	2010-2023	-1,16* (-1,87 a -0,46); p<0,001	-1,16* (-1,87 a -0,46); p<0,001
Minas Gerais	2010-2023	-3,4* (-3,79 a -2,99); p<0,001	-3,4* (-3,79 a -2,99); p<0,001	2010-2023	-2,76* (-3,1 a -2,42); p<0,001	-2,76* (-3,1 a -2,42); p<0,001
Paraná	2010-2023	-4,58* (-5,28 a -3,9); p<0,001	-4,58* (-5,28 a -3,9); p<0,001	2010-2016	-2,46 (-3,46 a 2,44); p=0,13	-3,41* (-4,13 a -2,7); p<0,001
				2016-2023	-4,22* (-8,81 a -3,38); p<0,001	
Paraíba	2010-2015	2,76* (0,12 a 8,77); p=0,04	-1,11* (-2,04 a -0,09); p=0,03	2010-2016	0,38 (-0,52 a 3,36); p=0,4	-0,72* (-1,21 a -0,24); p<0,01
	2015-2023	-3,45* (-6,18 a -2,17); p<0,001		2016-2023	-1,65* (-4,36 a -0,94); p<0,01	
Pará	2010-2012	10,33* (4,93 a 14,23); p<0,001	1,14* (0,67 a 1,64); p<0,001	2010-2023	1,03* (0,29 a 1,75); p<0,01	1,03* (0,29 a 1,75); p<0,01
	2012-2023	-0,45* (-1,01 a -0,02); p=0,04				
Pernambuco	2010-2018	-2,18 (-10,85 a 5,07); p=0,19	-3,64* (-5,67 a -2,21); p<0,001	2010-2018	-1,08 (-2,87 a 2,72); p=0,42	-2,24* (-4,18 a -0,85); p<0,01
	2018-2021	-13,12 (-18,92 a 5,42); p=0,14		2018-2021	-14,51* (-20,07 a -6,28); p=0,03	
	2021-2023	5,98 (-9,75 a 19,08); p=0,37		2021-2023	14,09 (-3,03 a 27,69); p=0,09	
Piauí	2010-2023	-0,68 (-1,79 a 0,42); p=0,22	-0,68 (-1,79 a 0,42); p=0,22	2010-2012	4,97 (-0,73 a 11,71); p=0,16	-0,29 (-1,42 a 0,57); p=0,26
				2012-2023	-1,22* (-5,65 a -0,47); p=0,04	
Rio Grande do Norte	2010-2023	-0,07 (-1,21 a 1,06); p=0,91	-0,07 (-1,21 a 1,06); p=0,91	2010-2023	0,88 (-0,09 a 1,81); p=0,07	0,88 (-0,09 a 1,81); p=0,07
Rio Grande do Sul	2010-2023	-3,97* (-4,85 a -3,1); p<0,001	-3,97* (-4,85 a -3,1); p<0,001	2010-2023	-3,25* (-4,04 a -2,48); p<0,001	-3,25* (-4,04 a -2,48); p<0,001
Rio de Janeiro	2010-2019	-1,11 (-2 a 0,1); p=0,07	-3,71* (-4,33 a -3,04); p<0,001	2010-2016	-0,51 (-1,93 a 2,52); p=0,59	-2,84* (-3,47 a -2,15); p<0,001

	2019-2023	-9,3* (-13,62 a -6,68); p<0,001		2016-2023	-4,79* (-6,85 a -3,66); p<0,001	
Rondônia	2010-2015	3,57 (-0,23 a 12,03); p=0,06	-2,31* (-3,56 a -0,96); p<0,001	2010-2012	6,04 (-2 a 14,43); p=0,27	-1,65* (-3,07 a -0,58); p<0,01
	2015-2023	-5,81* (-9,22 a -4,02); p<0,001		2012-2023	-2,99* (-8,63 a -2,09); p=0,01	
Roraima	2010-2023	3,07 (-0,49 a 6,64); p=0,09	3,07 (-0,49 a 6,64); p=0,09	2010-2023	2,61* (0,24 a 4,97); p=0,03	2,61* (0,24 a 4,97); p=0,03
Santa Catarina	2010-2023	-3,29* (-3,84 a -2,74); p<0,001	-3,29* (-3,84 a -2,74); p<0,001	2010-2023	-2,73* (-3,33 a -2,13); p<0,001	-2,73* (-3,33 a -2,13); p<0,001
São Paulo	2010-2023	-2,14* (-3,01 a -1,26); p<0,001	-2,14* (-3,01 a -1,26); p<0,001	2010-2023	-1,53* (-2,27 a -0,77); p<0,001	-1,53* (-2,27 a -0,77); p<0,001
Sergipe	2010-2023	-1,65* (-3 a -0,29); p=0,02	-1,65* (-3 a -0,29); p=0,02	2010-2023	-1,21* (-2,35 a -0,1); p=0,03	-1,21* (-2,35 a -0,1); p=0,03
Tocantins	2010-2023	-1,03* (-1,96 a -0,09); p=0,03	-1,03* (-1,96 a -0,09); p=0,03	2010-2023	0,03 (-1,2 a 1,29); p=0,95	0,03 (-1,2 a 1,29); p=0,95
Região Norte	2010-2012	5,63* (0,26 a 10,8); p=0,02	0,22 (-0,6 a 0,9); p=0,58	2010-2017	1,51* (0,85 a 3,82); p<0,001	0,55* (0,07 a 1,04); p=0,04
	2012-2023	-0,74* (-3,34 a -0,26); p=0,01		2017-2023	-0,56 (-3,08 a 0,28); p=0,19	
Região Nordeste	2010-2016	0,42 (-0,61 a 2,4); p=0,41	-1,35* (-1,8 a -0,88); p<0,001	2010-2017	0,86* (0,2 a 1,79); p=0,02	-0,3 (-0,67 a 0,05); p=0,08
	2016-2023	-2,83* (-4,24 a -1,98); p<0,001		2017-2021	-3,51* (-5,34 a -1,99); p=0,02	
				2021-2023	2,23 (-0,89 a 4,89); p=0,16	
Região Centro-Oeste	2010-2016	10,97* (6,08 a 19,58); p=0,02	-0,31 (-2,29 a 1,9); p=0,94	2010-2023	-1,35 (-3,79 a 1,29); p=0,3	-1,35 (-3,79 a 1,29); p=0,3
	2016-2019	-20,72* (-26,81 a -8,97); p=0,04				
	2019-2023	0,8 (-7,5 a 19,41); p=0,62				
Região Sudeste	2010-2023	-2,55* (-3,21 a -1,88); p<0,001	-2,55* (-3,21 a -1,88); p<0,001	2010-2023	-2,05* (-2,48 a -1,62); p<0,001	-2,05* (-2,48 a -1,62); p<0,001

Região Sul	2010-2023	-4,54* (-6,64 a -2,28); p<0,001	-4,54* (-6,64 a -2,28); p<0,001	2010-2023	-3,21* (-3,74 a -2,67); p<0,001	-3,21* (-3,74 a -2,67); p<0,001
Brasil	2010-2016	-1,29 (-2,36 a 4,28); p=0,25	-2,31* (-3,07 a -1,56); p<0,001	2010-2023	-1,68* (-2,16 a -1,2); p<0,001	-1,68* (-2,16 a -1,2); p<0,001
	2016-2023	-3,17* (-7,98 a -2,3); p<0,01				

UF: Unidade da Federação; IC 95%: Intervalo de Confiança de 95%; *estatisticamente significativo

Tabela S5. Percentual de variação anual (APC) das taxas de mortalidade de 30 a 69 anos padronizadas por idade(por 100 mil habitantes), segundo sexo e agravo – Brasil (2010-2023).

**Agravo**	**Região**	**Feminino**		**Masculino**	
		**Período**	**APC (IC 95%) p valor**	**Período**	**APC (IC 95%) p valor**
**DCV**	Norte	2010-2023	-1,51* (-1,97 a -1,06); p<0,001	2010-2020	-0,86 (-2,36 a 0,14); p=0,06
				2020-2023	0,83 (-0,69 a 3,01); p=0,45
	Nordeste	2010-2023	-2,03* (-2,44 a -1,63); p<0,001	2010-2017	-0,69 (-1,11 a 1,13); p=0,16
				2017-2021	-2,11* (-3,79 a -1,21); p=0,02
				2021-2023	2,75 (-0,44 a 5,09); p=0,08
	Centro-Oeste	2010-2019	-3,57* (-7 a -2,64); p=0,03	2010-2019	-2,76* (-3,54 a -2,33); p<0,001
		2019-2023	-0,45 (-2,87 a 4,31); p=0,69	2019-2023	0,18 (-1,25 a 2,83); p=0,79
	Sudeste	2010-2023	-2,39* (-2,82 a -1,96); p<0,001	2010-2020	-2,24* (-3,86 a -1,83); p<0,01

				2020-2023	0,44 (-1,6 a 3,63); p=0,73
	Sul	2010-2019	-4,01* (-6,84 a -3,14); p<0,01	2010-2020	-3,08* (-4,16 a -2,58); p<0,001
		2019-2023	0,07 (-2,57 a 5,7); p=0,9	2020-2023	1,45 (-1,44 a 5,3); p=0,31
	Brasil	2010-2020	-2,76* (-4,27 a -2,36); p<0,001	2010-2020	-2,08* (-2,83 a -1,76); p<0,001
		2020-2023	-0,28 (-2,21 a 2,48); p=0,79	2020-2023	0,6 (-1,18 a 3,11); p=0,51
**DCBV**	Norte	2010-2023	-2,41* (-3,02 a -1,79); p<0,001	2010-2014	-0,73 (-1,77 a 2,8); p=0,38
				2014-2020	-3,05* (-5,66 a -2,41); p<0,001
				2020-2023	1,78 (-0,76 a 5,46); p=0,15
	Nordeste	2010-2020	-3,51* (-5,24 a -2,95); p<0,001	2010-2021	-2,08 (-4,53 a 1,25); p=0,07
		2020-2023	-1,14 (-3,17 a 1,75); p=0,39	2021-2023	1,53 (-2,21 a 3,99); p=0,73
	Centro-Oeste	2010-2020	-3,49 (-6,57 a 0,41); p=0,06	2010-2019	-3,34* (-5,71 a -2,73); p<0,01
		2020-2023	-0,92 (-4,18 a 2,85); p=0,48	2019-2023	-0,76 (-2,54 a 2,87); p=0,64
	Sudeste	2010-2020	-3,49* (-5,69 a -2,27); p=0,02	2010-2014	-5,28* (-8,6 a -3,53); p<0,001
		2020-2023	-0,7 (-3,17 a 2,56); p=0,47	2014-2023	-1,94* (-2,43 a -0,74); p=0,02
	Sul	2010-2020	-4,16* (-6,95 a -3,38); p<0,01	2010-2019	-4,01* (-7,95 a -2,97); p=0,02
		2020-2023	0,81 (-3,1 a 6,16); p=0,75	2019-2023	0,32 (-2,74 a 6,42); p=0,85
	Brasil	2010-2020	-3,54* (-4,25 a -3,2); p<0,001	2010-2019	-3,15* (-5,17 a -2,63); p<0,01

		2020-2023	-0,61 (-2,55 a 2,01); p=0,52	2019-2023	-0,38 (-2,13 a 3,14); p=0,62
**DH**	Norte	2010-2023	-1,34* (-2,58 a -0,09); p=0,04	2010-2023	-0,04 (-1,14 a 1,07); p=0,95
	Nordeste	2010-2018	-2,92 (-10,98 a 10,19); p=0,14	2010-2018	-2,38 (-6,05 a 0,2); p=0,05
		2018-2021	5,79 (-7,24 a 10,58); p=0,31	2018-2021	9,52 (-3,13 a 13,64); p=0,08
		2021-2023	-7,15 (-18,12 a 4,77); p=0,31	2021-2023	-8,04 (-15,64 a 3,23); p=0,11
	Centro-Oeste	2010-2023	-2,53* (-3,54 a -1,5); p<0,001	2010-2023	-1,24 (-2,69 a 0,2); p=0,09
	Sudeste	2010-2015	-6,45* (-10,35 a -3,92); p<0,01	2010-2015	-5,09* (-8,08 a -2,95); p<0,001
		2015-2021	3,01* (1,31 a 8,11); p=0,01	2015-2021	4,34* (2,89 a 8,3); p<0,01
		2021-2023	-7,61* (-13,27 a -1,45); p=0,01	2021-2023	-7,53* (-12,38 a -1,71); p<0,01
	Sul	2010-2015	-4,66* (-14,26 a -0,71); p=0,02	2010-2019	0,03 (-3,29 a 1,07); p=0,82
		2015-2023	2,14 (-0,06 a 10,71); p=0,06	2019-2023	5,27* (1,96 a 12,17); p<0,001
	Brasil	2010-2014	-5,78* (-13,28 a -1,85); p<0,01	2010-2015	-4,04* (-9,77 a -1,23); p=0,02
		2014-2023	0,37 (-0,97 a 5,5); p=0,44	2015-2021	3,42* (0,89 a 8,99); p=0,04
				2021-2023	-5,11 (-11,8 a 1,33); p=0,11
**DIC**	Norte	2010-2023	-1,18* (-1,76 a -0,59); p<0,001	2017-2023	1,18* (0,34 a 5,48); p=0,02
				2010-2023	-1,07* (-5,58 a 0); p=0,05
	Nordeste	2010-2018	-1,01 (-4,45 a 1,35); p=0,15	2018-2021	0,35 (-0,11 a 1,07); p=0,13

	2018-2021	-6,49 (-9,22 a 1,93); p=0,11	2021-2023	-5,4* (-6,73 a -3,48); p<0,001
	2021-2023	2,61 (-4,54 a 7,68); p=0,32	2010-2017	4,51* (1,45 a 7,22); p<0,01
Centro-Oeste	2010-2023	-1,9* (-2,53 a -1,27); p<0,001	2010-2018	-1,16* (-1,63 a -0,69); p<0,001
Sudeste	2010-2023	-2,59* (-3,15 a -2,02); p<0,001	2010-2023	-2,02* (-2,45 a -1,59); p<0,001
Sul	2010-2023	-4,04* (-4,73 a -3,35); p<0,001	2010-2023	-3,25* (-3,82 a -2,69); p<0,001
Brasil	2010-2023	-2,58* (-3,08 a -2,07); p<0,001	2010-2023	-1,77* (-2,15 a -1,38); p<0,001

IC 95%: Intervalo de Confiança de 95%; *estatisticamente significativo
