# Supplementary material for: Tendência Temporal da Mortalidade por Doenças Cardiovasculares: Estudo Ecológico das Regiões e Unidades da Federação do Brasil, 2010-2023
Source: Arq Bras Cardiol. 2026 Sep 10;123(8):e20260087. [Article in Portuguese] doi: 10.36660/abc.20260087 (PMC13671139; doi:10.36660/abc.20260087)
Supplement: Translation

# Translation: Time Trends in Mortality from Cardiovascular Diseases: An Ecological Study of Brazil’s Regions and States, 2010-2023

**DOI:** 10.36660/abc.20260087i

**Authors:** José Francisco Kerr Saraiva, Ana Clara Paixão Campos, Gisele O’Dwyer, Anna Carolina Pinho da Silva, Bruna Bornhofen Barros, Monique Alves Padilha, Fernanda Cristina da Silva de Lima, Catia Cristina Martins Oliveira

**Affiliations:** 1 Brazil Pontifícia Universidade Católica de Campinas, Campinas, SP – Brazil; 2 Brazil Centro de Integração de Dados e Conhecimentos para Saúde (CIDACS), Fundação Oswaldo Cruz, Salvador, BA – Brazil; 3 Brazil Escola Nacional de Saúde Pública Sérgio Arouca – ENSP/Fiocruz, Rio de Janeiro, RJ – Brazil; 4 Brazil Centro Universitário Serra dos Órgãos (UNIFESO), Teresópolis, RJ – Brazil; 5 Brazil Universidade Federal do Rio de Janeiro – Instituto de Estudos em Saúde Coletiva (IESC), Rio de Janeiro, RJ – Brazil; 6 Brazil Ministério da Saúde, Secretaria de Informação e Saúde Digital (SEIDIGI), Brasília, DF – Brazil; 7 Brazil Centro de Desenvolvimento de Tecnologias em Saúde (CDTS), Fundação Oswaldo Cruz – Rio de Janeiro, RJ – Brazil

**Keywords:** Cardiovascular Diseases, Mortality, Time Series Studies, Epidemiology

## Abstract

**Background:**

Death from cardiovascular diseases (CVD) represents an essential marker for evaluating the progress and limitations of public policies, especially in territories with greater vulnerability.

**Objective:**

To analyze mortality from CVD, hypertensive diseases (HD), ischemic heart disease (IHD), and cerebrovascular diseases (CEVD) by sex, in Brazil, its regions, and Federative Units, between 2010 and 2023.

**Methods:**

Time series study with mortality data obtained from DATASUS. Rates were standardized by age, and the temporal trend was assessed using the Joinpoint regression model, with the calculation of annual percent change (APC) and the Average Annual Percentage Change (AAPC) for the entire period. A 95% confidence interval and a significance level of 5% were considered.

**Results:**

Between 2010 and 2023, there were 4,991,937 deaths from CVD in Brazil (52.50% in men). Mortality from CVD decreased in men (Federal District: -3.58%, p<0.001; South: -2.23%, p<0.001), but increased in Amazonas (1.04%, p<0.01) and Amapá (1.54%, p<0.01). IHD increased in men in Amapá (5.21%, p<0.001) and Roraima (2.61%, p=0.03). HD increased in Bahia (men: 1.38%, p=0.03; women: 1.50%, p=0.04) and Rio Grande do Sul (men: 1.93%, p<0.001; women: 1.86%, p<0.01). CEVD mortality shows a downward trend in most states. Premature mortality from HD increased among men in the South (+1.61%, p<0.001).

**Conclusion:**

In Brazil, despite advances in the prevention and treatment of CVD, improvements have not been equitable: strong regional and socioeconomic inequalities persist, with higher mortality rates among vulnerable populations.

## Introduction

Cardiovascular diseases (CVDs) remain one of the main challenges to global health, ranking as the leading cause of mortality and one of the biggest contributors to disability and loss of healthy years of life worldwide since 1990.^1,2^ This scenario is driven by changes in risk factors – notably body mass index, which has the strongest association with CVD^3^ – and by population growth and aging.^2^ In 2022, CVDs caused approximately 19.8 million deaths worldwide, a third of the global total, resulting in a loss of 396 million years of life.^2^ A relevant fact is that about 34% of these deaths were premature (in people aged 30 to 69 years), and are considered largely preventable through interventions already available, such as the control of risk factors.^1-5^

In Brazil, since 1990, CVDs have led the mortality statistics among non-communicable chronic diseases (NCDs).^6^ Among these, coronary artery disease remains the leading cause of death, followed by Stroke.^6^ These diseases and their complications have a high impact on lost productivity at work and reduced family income, affecting socioeconomically vulnerable populations more intensely, whose limited access to quality health services deepens the cycle of disparities.^7^

In addition to individual factors, territory is a central dimension in the production of health inequalities in Brazil. The country presents historical regional heterogeneities in income, education, urban infrastructure, and the provision of health services, which are reflected in social and health indicators.^8^ Studies indicate that residents of less developed regions have greater exposure to socioeconomic vulnerabilities and worse health indicators.^9,10^

In the context of public policies, in 2011, the Ministry of Health launched the Strategic Action Plan to Combat Chronic Non-Communicable Diseases (NCDs) in Brazil, 2011-2022, intending to promote the development and implementation of effective, integrated, sustainable, and evidence-based public policies for the prevention and control of NCDs. In addition, the Cardiovascular Health Strategy in Primary Health Care was implemented in 2022, aligned with the global HEARTS initiative (WHO/PAHO), aiming to strengthen the early detection and control of risk factors such as hypertension and diabetes. However, the advancement of these policies faces social, organizational, and economic challenges hindering their sustainability and impact on health indicators.

Given this scenario, the disaggregated analysis of mortality by regions and Federative Units (UF) becomes a strategic marker for evaluating the effectiveness of public policies that have been developed in the country, especially due to the profound socioeconomic and structural inequities, contributing to identifying disparities in life expectancy and causes of death, directing resources more fairly, and preventing avoidable deaths.

Considering the high mortality burden imposed by CVD and its significant direct (hospitalization and procedures) and indirect (loss of productivity and premature death) costs, this study aimed to analyze the temporal trend of age-standardized CVD mortality rates and their main subgroups – hypertensive diseases (HD), ischemic heart diseases (IHD) and cerebrovascular diseases (CEVD) – in Brazil, regions and Federative Units, from 2010 to 2023.

## Methods

### Study design

Ecological time-series study, analyzing mortality from CVD in Brazil, its regions, and Federative Units, according to sex, from 2010 to 2023, using deaths registered by place of residence as the unit of analysis.

### Source of information

Data on the causes of death were obtained from the Mortality Information System (SIM) website, maintained by the Department of Informatics of the Unified Health System (DATASUS) of the Ministry of Health. Records of deaths from CVD were included, defined as the underlying cause under codes I00-I99 (Diseases of the Circulatory System) of the Tenth Revision of the International Statistical Classification of Diseases and Related Health Problems (ICD-10). The analysis also focused on three subgroups of relevance to public health: HD (I10–I15), IHD (I20–I25), and CEVD (I60–I69).

### Statistical analysis

Annual estimates of the resident population in Brazil, its regions, and states were obtained from the DATASUS website, which uses official data from the Brazilian Institute of Geography and Statistics (IBGE) Census for the year 2010, along with intercensal projections, calculated by fitting a 3rd degree polynomial function.^11^

The variables used were sex (male and female), age group in years (< 20 years (excluding 20), 20 to 29 years, 30 to 39 years, 40 to 49 years, 50 to 59 years, 60 to 69 years, and ≥70 years), race/color (white, black, brown, yellow, and indigenous), schooling in years (none, 1 to 3 years, 4 to 7 years, 8 to 11 years), region of residence (North; Northeast; South; Southeast; Midwest), the 26 Brazilian states (Acre, Alagoas, Amazonas, Amapá, Bahia, Ceará, Espírito Santo, Goiás, Maranhão, Minas Gerais, Mato Grosso do Sul, Mato Grosso, Pará, Paraíba, Pernambuco, Piauí, Paraná, Rio de Janeiro, Rio Grande do Norte, Rondônia, Rio Grande do Sul, Santa Catarina, Sergipe, São Paulo, Tocantins) and the Federal District. The mortality rate per 100,000 inhabitants was calculated, standardized by age, considering the variables sex, age group, year of death (between 2010 and 2023), region, and Federal Unit of residence.

The study initially conducted descriptive analyses of sociodemographic variables considering the number and proportion of deaths from CVD in the period from 2010 to 2023. Mortality rates were standardized by age using the direct method, taking as a reference the world standard population proposed by Segi.^12^ This procedure is essential to minimize the bias caused by variations in the age distribution, isolating the real effect of the risk of death from CVD. For the calculation of the standardized mortality rate, data filled in as ignored were excluded from the analyses.

Temporal trends were estimated using the Joinpoint regression model. This methodology aims to identify the model that best describes the behavior of rates over time, using connected line segments, with or without “inflection points” (joinpoints). In this approach, mortality rates (dependent variable) were transformed on a logarithmic scale, while the years of the study period were considered as the independent variable.^13^

The choice of the final model was based on Monte Carlo permutation tests, adopting the one that presented the highest number of joinpoints with statistical significance (p<0.05).^13^ After defining the selected model, the Annual Percent Change (APC) and the Average Annual Percent Change (AAPC) were calculated, with their respective confidence intervals and p-values. The AAPC values presented here describe the direction (increase, decrease, stability) and intensity of the changes observed over time.^13,14^ The following methodological criteria were adopted: a minimum historical series of 10 years and exclusion of zero values in the time series, since the calculation of annual percentage changes (APC) and annual average changes (AAPC) uses logarithmic transformation, making the logarithm of zero mathematically undefined, which could compromise the analysis with errors. Statistical analyses were conducted using R software (version 4.5.1)^15^ and the Joinpoint Regression Program (version 5.4.0),^16^ and the tables and figures were developed in Microsoft Excel.

The study did not require ethical approval, as it used public mortality data without identifying the participants.

## Results

From 2010 to 2023, there were 4,991,937 deaths from CVD in Brazil, the majority in males (52.50%). Among these deaths, the three main CVDs were responsible for a significant portion: IHD caused 1,561,034 deaths (approximately 31.30% of all CVD deaths), Cerebrovascular Disease led to 1,418,725 deaths (approximately 28.40%), and Hypertensive Disease resulted in 749,392 deaths (approximately 15%). Together, these three groups accounted for almost three-quarters of all CVD-related deaths during this period (Central illustration). The proportion of deaths increased progressively with age in both sexes. Men had higher percentages in most age groups, except among individuals aged 70 or older, where women predominated (68.60%). White women accounted for 54.35% of deaths. The highest proportions of deaths in women occurred in the Southeast (47.26%) and Northeast (26.40%), and low education (“None” and “1 to 3 years”) also concentrated higher percentages of deaths among women (47.77%) (Table 1).

**Central Illustration:** : Time Trends in Mortality from Cardiovascular Diseases: An Ecological Study of Brazil’s Regions and States, 2010-2023

**Table 1:** – Sociodemographic characterization of deaths from cardiovascular diseases in Brazil, 2010-2023 (n=4,991,937)

Variable	Male n=2,620,654 (52.50%)	Female n=2,370,766 (47.49%)	Ignored n=517 (0,01%)
	n (%)	n (%)	n (%)
**Age range**			
Under 20 years old	11,773 (0.45)	8,969 (0.38)	4 (0.77)
20 to 29 years old	21,430 (0.82)	12,507 (0.53)	2 (0.39)
30 to 39 years old	57,266 (2.19)	35,997 (1.52)	10 (1.93)
40 to 49 years old	155,738 (5.94)	101845 (4.30)	11 (2.13)
50 to 59 years old	355,198 (13.55)	209440 (8.83)	39 (7.54)
60 to 69 years old	575,369 (21.96)	373,668 (15.76)	70 (13.54)
70+ years	1,438,467 (54.89)	1,626,386 (68.60)	246 (47.58)
Ignored	5,413 (0.21)	1,954 (0.08)	135 (26.11)
**Race/color**			
White	1,322,820 (50.48)	1,288,530 (54.35)	72 (13.93)
Black	236,083 (9.01)	201,989 (8.52)	23 (4.45)
Brown	954,325 (36.42)	780,702 (32.93)	113 (21.86)
Yellow	15,563 (0.59)	14,353 (0.61)	4 (0.77)
Indigenous	5,119 (0.20)	4,506 (0.19)	8 (1.55)
Ignored	86,744 (3.31)	80,686 (3.40)	297 (57.45)
**Education (years)**			
None	425,034 (16.22)	549,058 (23.16)	45 (8.70)
1 to 3 years	632,581 (24.14)	583,407 (24.61)	49 (9.48)
4 to 7 years	564,843 (21.55)	450,234 (18.99)	27 (5.22)
8 to 11 years	374,178 (14.28)	260,483 (10.99)	21 (4.06)
12 years or older	137,063 (5.23)	85,157 (3.59)	10 (1.93)
Ignored	486,955 (18.58)	442,427 (18.66)	365 (70.60)
**Region**			
North	147,846 (5.64)	110,403 (4.66)	38 (7.35)
Northeast	678,805 (25.90)	625,858 (26.40)	152 (29.40)
Midwest	178,827 (6.82)	141,063 (5.95)	49 (9.48)
Southeast	1,216,092 (46.40)	1,120,534 (47.26)	240 (46.42)
South	399,084 (15.23)	372,908 (15.73)	38 (7.35)

The analysis of time segments for CVD and subgroups (Tables S1 to S4) revealed greater oscillation (more joins) for CVD in the North region; for CEVD in the North and Southeast; For HD in the Southeast (3 segments) and Midwest; and for IHD in the North, Northeast, and Midwest (3 segments for women). This reveals patterns of instability that vary according to the cause and region. In the segmented analysis by region for the population aged 30 to 69 years (Table S5), distinct patterns were observed among the conditions, with a clear impact of the pandemic period on the trajectories. CVD and CEVD showed decreases until 2019-2020 in all regions, led by the South and Southeast, followed by stabilization or slight reversal during the pandemic period. HD exhibited a more irregular trajectory, with emphasis on the Southeast (decrease until 2015, increase until 2021, and a new decrease) and for men in the South, who had an increase in the period 2019-2023 (+5.27%). IHD was the most stable and favorable condition, with widespread declines, except for the increase among men in the North of the country from 2017 to 2023 (+1.18%).

The following trend analyses were based on AAPC values from the Joinpoint regression model.

### Mortality from CVD

Figure 1 shows a significant decrease in CVD mortality in Brazil (men: -1.52%; p<0.001 and women: -2.01%; p<0.001), with the South region showing the greatest reductions (-2,23%; p<0.001 and -2.63%; p<0.001, respectively). In contrast, the Northern region revealed opposite situations: while Amapá registered an increase for both sexes (+1.54%; p<0.01 for men and +2.05%; p=0.03 for women) and Amazonas only for men (+1.04%; p<0.01), states like Rondônia and Tocantins stood out for significant declines in the female population (-1.68%; p<0.001 and -2.29%; p<0.001, respectively) — differences that reinforce the regional heterogeneity in the Brazilian territory.

**Figure 1:** – Trends in mortality rates from cardiovascular diseases and hypertensive diseases (HD) standardized by age (per 100,000 population), by sex, Brazil, regions, states, and Federal District, 2010 – 2023. *AAPC statistically significant (p<0.05).

### Mortality from HD

Still in Figure 1, the analysis of mortality from HD revealed equally heterogeneous patterns. At the regional level, only the Southeast showed a significant decrease for women (-1.10%; p<0.01), while in the other regions and in Brazil, the trend was one of stability for both sexes. In contrast to the predominantly stable regional pattern, some states exhibited significant variations. In the North region, increases were notable in Amazonas (men: +3.28%; p<0.01 and women: +2.26%; p<0.01) and Amapá (women: +3.92%; p=0.04), contrasting with decreases in Acre (women: -4.40%; p=0.04), Roraima (men: -3.76%; p=0.01), Rondônia (women: -1.67%; p=0.01) and Tocantins (women: -1.71%; p=0.03). In the Northeast, unlike most states, Bahia showed an increase for both sexes (+1.38%; p=0.03 in men and +1.50%; p=0.04 in women). In the Central-West region, decreases predominated, especially among men in the Federal District (-1.89%; p=0.01) and Mato Grosso do Sul (-2.41%; p=0.01). In the Southeast, Rio de Janeiro stood out with a reduction for both sexes (-1.28%; p<0.001 in men and -1.49%; p<0.001 in women). Finally, in the South region, the most significant increase was observed in Rio Grande do Sul (men: +1.93%; p<0.001 and women: +1.86%; p<0.01).

### Mortality from IHD

Figure 2 shows that the trend in mortality from IHD revealed a national contrast between the sexes: an increase among men and a decrease among women. In the North region, the general pattern was one of increase, driven by male growth. Notable cases were the increases in Amapá (+5.21; p<0.001) and Roraima (+2.61; p=0.03) among men, and in Pará among women (+1.14; p<0.001). In the Northeast, the reduction restricted to women masked internal heterogeneity: Alagoas registered one of the largest increases (+2.62; p=0.01) in the female population in Brazil, contrasting with the significant decreases observed in Pernambuco (-3.64; p<0.001). In the Central-West region, the declines were moderate and restricted to Mato Grosso and Mato Grosso do Sul. The Southeast, however, showed significant and widespread reductions for both sexes, with Rio de Janeiro (men: -2.84%; p<0.001 and women: -3.71%; p<0.001) leading the declines—these being among the most pronounced in the country. The South, in turn, was the region with the greatest national reduction, particularly women in Paraná (-4.58%; p<0.001), who experienced the largest declines among all regions.

**Figure 2:** – Trends in mortality rates from Ischemic Heart Disease (IHD) and Cerebrovascular Disease (CEVD) standardized by age (per 100,000 population), by sex, Brazil, regions, states, and Federal District, 2010 – 2023. *AAPC statistically significant (p<0.05).

### Mortality from CEVD

For CEVD (Figure 2), a generalized and significant decrease in mortality trends was observed for Brazil and all regions, in both sexes, with women showing more significant reductions. The South region led the decreases, while the North, despite the favorable trend, exhibited the most modest declines in the country. In the North region, a scenario is observed in which most states showed a decrease in mortality rate trends, especially Rondônia (-1.75%; p<0.01 for men and -3.29%; p<0.001 for women) and Tocantins (-1.94%; p<0.001 for men and -3.03%; p<0.001 for women). In the Northeast, except for the male population of Bahia, all other states showed a downward trend in CVD mortality rates in both sexes. All states in the Central-West showed a decline in mortality rates. The Federal District had the largest reduction among women (-4.55%; p<0.001), and Mato Grosso had the largest drop among men (-3.87%; p<0.001). In the Southeast, all states reduced mortality rates for both sexes, with Espírito Santo (-4.07%; p<0.001 for men and -4.17%; p<0.001 for women) leading the most significant decreases in the country. All federative units in the South of the country showed a significant reduction in the mortality rate, with particular emphasis on the female population of Rio Grande do Sul (-4.26%; p<0.001).

### Mortality in the population aged 30 to 69 years

Premature mortality from CVD (Table 2) decreased in Brazil, with sharper drops among women (-2.19%), led by the South (-2.77%) and Central-West (-2.62%) regions. For HD, the national decrease was significant only among women (-1.56%), particularly in the Central-West, Southeast, and North regions. In IHD, the reduction was significant in both sexes, but almost twice as high among women (-2.58% vs. -1.77% in men), with the South leading the declines. In CEVD, the decreases were also significant for both sexes, more pronounced among women (-2.87%), with a greater female decline in the South and a greater male decline in the Southeast. In all cause groups, women showed greater reductions than men in practically all regions.

**Table 2:** – Trend in premature mortality rate (30 to 69 years) due to cardiovascular diseases and subgroups – HD, IHD, and CEVD – standardized by age (per 100,000 population), by sex, Brazil, regions, 2010 – 2023

Region		Male				Female			
		CVD	HD	IHD	CEVD	CVD	HD	IHD	CEVD
North	AAPC (CI95%)	-0.47* (-0.81 to -0.22)	-0.04 (-1.14 to 1.07)	0.14 (-0.54 to 0.84)	-1.24* (-1.67 to -0.75)	-1.51* (-1.97 to -1.06)	-1.34* (-2.58 to -0.09)	-1.18* (-1.76 to -0.59)	-2.41* (-3.02 to -1.79)
Northeast	AAPC (CI95%)	-0.61* (-0.99 to -0.29)	-0.67 (-1.89 to 0.56)	-0.39* (-0.73 to -0.01)	-1.53* (-2.15 to -1.17)	-2.03 * (-2.44 to -1.63)	-1.65 (-3.46 to 0.69)	-1.76* (-2.66 to -1.16)	-2.97* (-3.41 to -2.65)
Midwest	AAPC (CI95%)	-1.87* (-2.23 to -1.52)	-1.24 (-2.69 to 0.20)	-1.16* (-1.63 to -0.69)	-2.55* (-3.09 to -2.11)	-2.62* (-3.29 to -2.11)	-2.53* (-3.54 to -1.50)	-1.9 * (-2.53 to -1.27)	-2.91* (-3.57 to -2.43)
Southeast	AAPC (CI95%)	-1.63* (-2.09 to -1.24)	-1.24* (-1.99 to -0.54)	-2.02* (-2.45 to -1.59)	-2.98* (-3.29 to -2.54)	-2.39 * (-2.82 to -1.96)	-2.38* (-3.26 to -1.60)	-2.59* (-3.15 to -2.02)	-2.85* (-3.38 to -2.50)
South	AAPC (CI95%)	-2.05* (-2.59 to -1.66)	1.61* (0.71 to 2.42)	-3.25* (-3.82 to -2.69)	-2.69* (-3.53 to -2.07)	-2.77* (-3.51 to -2.19)	-0.53 (-1.85 to 0.97)	-4.04* (-4.73 to -3.35)	-3.04* (-3.87 to -2.46)
Brazil	AAPC (CI95%)	-1.47* (-1.81 to -1.18)	-0.84 (-1.88 to 0.10)	-1.77* (-2.15 to -1.38)	-2.3* (-2.75 to -1.98)	-2.19* (-2.61 to -1.89)	-1.56* (-2.49 to -0.41)	-2.58* (-3.08 to -2.07)	-2.87* (-3.23 to -2.62)

CI 95%: 95% Confidence Interval; AAPC: Average annual percentage change; *statistically significant. CVD: cardiovascular diseases; HD: hypertensive diseases; IHD: ischemic heart disease; CEVD: cerebrovascular diseases

## Discussion

This study observed a higher proportion of deaths among individuals with lower levels of education, demonstrating that unfavorable socioeconomic conditions remain associated with worse cardiovascular outcomes.^17^ Evidence from other research shows that low education is related to greater exposure to cardiovascular risk factors, including inadequate diet, less physical activity, poorer housing conditions, higher prevalence of obesity and hypertension, as well as barriers to access and continuity of care in health services.^18,19^In Brazil, cardiovascular mortality should be understood not only as a result of individual biomedical factors, but also as an expression of the social and structural inequalities historically present in the country^19,20^—where their persistence reinforces the role of the social determinants of health in this burden. In addition to education, the distribution of deaths according to race/color reinforces the relevance of racial inequities in cardiovascular mortality, even though some records contain missing information, especially in historically vulnerable populations. National studies demonstrate that the black population have greater exposure to cardiovascular risk factors, as well as significant difficulties in accessing longitudinal health care.^20-28^ It is important to emphasize that the high proportion of records missing race/color, observed in this study, highlights persistent limitations in the quality of information, indicating the need for greater rigor in its completion.

From a trend analysis perspective, the findings of this study demonstrate that, although Brazil has shown an overall reduction in mortality from CVD between 2010 and 2023, this epidemiological transition occurred heterogeneously across regions, especially when analyzed according to sex and subgroups of CVDs. In general, the most significant reductions observed in the South, Southeast, and Midwest regions contrast with less favorable trends recorded in states in the North and Northeast regions, suggesting that national advances coexist with persistent structural, social, and territorial inequalities in access to prevention, diagnosis, and treatment of CVD. These results are consistent with other national and international studies that demonstrate a progressive reduction in cardiovascular mortality in several countries in recent decades, but accompanied by significant intra- and inter-regional disparities.^23-28^

The differences between men and women also deserve highlighting. Cardiovascular mortality remained systematically higher among men, a pattern already described in the national and international literature.^18,29^ This behavior may reflect lower demand for preventive care, lower therapeutic adherence, and a higher prevalence of behavioral risk factors in this group, such as smoking, excessive alcohol consumption, and physical inactivity during leisure time.^18,19^ Among women, although overall mortality was lower, CVDs often present with atypical clinical manifestations and lower diagnostic recognition, which can delay appropriate management and contribute to worse outcomes at older ages.^29,30^ These findings reinforce the need for preventive and care strategies, especially within the scope of primary health care, that are sensitive to gender differences throughout the life course.

When analyzing specific CVD subgroups, a significant reduction in mortality from IHD and cerebrovascular disease (CVD) was observed, especially in the South and Southeast regions. These results are consistent with advances observed in the organization of the cardiovascular and cerebrovascular care pathway in Brazil, including greater availability of effective pharmacological therapies, expansion of clinical protocols, expansion of specialized units, and gradual incorporation of care technologies, such as telemedicine and care regulation systems.^30-32^

In contrast, mortality from HD showed a trend of stability or increase in some states, particularly in the North and Northeast regions, suggesting that, although the country has advanced in the management of acute cardiovascular events, challenges related to the longitudinal control of hypertension and the continuous management of cardiovascular risk factors persist.^33^

Hypertension remains one of the main modifiable risk factors for CVD and stroke, requiring sustained strategies of screening, clinical monitoring, and therapeutic adherence over time.^33^ In this context, primary health care (PHC) plays a central role in coordinating care, providing longitudinal follow-up, and preventing cardiovascular complications, especially in socially vulnerable populations.

Data from the National Survey on Access, Use, and Promotion of the Rational Use of Medicines (PNAUM) demonstrated high availability of antihypertensive medications in the country, with access close to 98% for the treatment of hypertension after the expansion of public pharmaceutical policies. The expansion of access to medications for this group of chronic conditions, offered by the Popular Pharmacy Program, may also have contributed to the patterns observed in this study.^34^

In addition, multidisciplinary care in PHC, involving nursing, pharmacy, physical educators, nutrition, and community health agents, can promote greater therapeutic adherence, blood pressure control, and reduction of cardiovascular complications, particularly in territories with more structured care networks.^35^

Although premature mortality from CVD decreased in Brazil between 2010 and 2023, the reductions occurred unevenly across regions. The smaller declines observed in the North and part of the Northeast indicate that the risk of premature death from CVD depends not only on expanding access to care, but also on the implementation of broader intersectoral public policies aimed at reducing social inequalities, promoting healthy eating, improving urban conditions, food security, and strengthening primary health care.^19,23,28^

Another relevant aspect for interpreting the observed regional inequalities refers to the historical heterogeneity of the quality of mortality information in Brazil. Regions such as the North and Northeast have presented, over the last few decades, higher proportions of underreporting of deaths, poorly defined causes, and less completeness of death certificates.^27,28^ Thus, some of the less favorable trends observed in some states may reflect not only real changes in cardiovascular mortality, but also gradual advances in the coverage and qualification of the Mortality Information System (SIM), including improvements in the coding of basic causes of death and greater capacity for epidemiological surveillance in the notification of diseases.

The limitations of this study should be considered when interpreting the results, as secondary data from the Mortality Information System (SIM) were used, which are subject to possible inconsistencies related to data completion, underreporting, and regional variability in information quality. Additionally, the COVID-19 pandemic period may have influenced the classification of underlying causes of death and the organization of health services, partially impacting the observed trends. The absence of individual clinical variables and more detailed information on access to health services also limits more robust causal interpretations. Despite these limitations, the study uses one of the main national mortality databases in the country, allowing for a comprehensive assessment of the temporal trends of CVDs in different Brazilian territorial contexts.

## Conclusions

Analysis of cardiovascular disease mortality in Brazil reveals that, although there have been advances in the prevention, diagnosis, and treatment of this group of diseases in recent decades, the benefits have not been distributed equitably among different population groups and territories. Significant regional and socioeconomic inequalities persist, with higher mortality rates concentrated in more vulnerable populations, characterized by lower education levels, lower income, poorer housing conditions, and greater difficulty in accessing timely health services. Thus, addressing the burden of CVD in the country requires not only expanding access to cardiovascular care but also strengthening macro-policies focused on health equity and the social determinants of CVDs. It is recommended that future research focus its investigations from an intersectional perspective (gender, race/color, class, and territory) to identify higher-risk subgroups and support policies for social protection and equitable access to cardiovascular health services.

## Supplemental Materials

Material Suplementar
